# Transcription Factor Activity Inference in Systemic Lupus Erythematosus

**DOI:** 10.3390/life11040299

**Published:** 2021-04-01

**Authors:** Raul Lopez-Dominguez, Daniel Toro-Dominguez, Jordi Martorell-Marugan, Adrian Garcia-Moreno, Christian H. Holland, Julio Saez-Rodriguez, Daniel Goldman, Michelle A. Petri, Marta E. Alarcon-Riquelme, Pedro Carmona-Saez

**Affiliations:** 1 Centre for Genomics and Oncological Research, Pfizer-University of Granada-Andalusian Regional Government, 18016 Granada, Spain; raul.lopez@genyo.es (R.L.-D.); daniel.toro@genyo.es (D.T.-D.); jordi.martorell@genyo.es (J.M.-M.); adrian.garcia@genyo.es (A.G.-M.); 2 Atrys Health S.A., 08025 Barcelona, Spain; 3 Faculty of Medicine, Institute of Computational Biomedicine, Heidelberg University, 69120 Heidelberg, Germany; christian.holland@bioquant.uni-heidelberg.de (C.H.H.); julio.saez@bioquant.uni-heidelberg.de (J.S.-R.); 4 Joint Research Center for Computational Biomedicine, Faculty of Medicine, RWTH Aachen University, 52062 Aachen, Germany; 5 Division of Rheumatology, Johns Hopkins University School of Medicine, Baltimore, MD 21205, USA; dgoldm11@jhmi.edu (D.G.); mpetri@jhmi.edu (M.A.P.); 6 Unit of Chronic Inflammatory Diseases, Institute of Environmental Medicine, Karolinska Institutet, 17177 Solna, Sweden; 7 Biostatistics, Department of Statistics, Faculty of Medicine, University of Granada, 18071 Granada, Spain

**Keywords:** transcription factor activity inference, clustering analysis, systemic lupus erythematosus, disease classification

## Abstract

Background: Systemic Lupus Erythematosus (SLE) is a systemic autoimmune disease with diverse clinical manifestations. Although most of the SLE-associated loci are located in regulatory regions, there is a lack of global information about transcription factor (TFs) activities, the mode of regulation of the TFs, or the cell or sample-specific regulatory circuits. The aim of this work is to decipher TFs implicated in SLE. Methods: In order to decipher regulatory mechanisms in SLE, we have inferred TF activities from transcriptomic data for almost all human TFs, defined clusters of SLE patients based on the estimated TF activities and analyzed the differential activity patterns among SLE and healthy samples in two different cohorts. The Transcription Factor activity matrix was used to stratify SLE patients and define sets of TFs with statistically significant differential activity among the disease and control samples. Results: TF activities were able to identify two main subgroups of patients characterized by distinct neutrophil-to-lymphocyte ratio (NLR), with consistent patterns in two independent datasets—one from pediatric patients and other from adults. Furthermore, after contrasting all subgroups of patients and controls, we obtained a significant and robust list of 14 TFs implicated in the dysregulation of SLE by different mechanisms and pathways. Among them, well-known regulators of SLE, such as STAT or IRF, were found, but others suggest new pathways that might have important roles in SLE. Conclusions: These results provide a foundation to comprehend the regulatory mechanism underlying SLE and the established regulatory factors behind SLE heterogeneity that could be potential therapeutic targets.

## 1. Introduction

Systemic Lupus Erythematosus (SLE) is a complex and multisystemic autoimmune disease characterized by the production of autoantibodies leading to chronic inflammation and organ damage. SLE is very heterogeneous, with possible affliction in almost any organ and diverse clinical manifestations including skin rashes, arthritis and renal failure [1]. SLE is known to be much more prevalent in females than in males and when it does occur in men tends to run a more severe course [2]. SLE patients usually suffer uncertain courses of flares, improvement and remission of disease activity, which makes this disease unpredictable. Disease activity can be measured by several indices, but one of the most accepted is the SLE Disease Activity Index (SLEDAI). It is measured by 24 laboratory and weighted clinical variables in nine organ systems including renal, skin and arthritis [3]. SLE is a challenge for researchers because of the difficulty of making an early diagnosis and the fact that the available drugs do not have a therapeutic effect for all patients [4].

With the development of genomics technologies, our knowledge about the pathogenesis of SLE and the underlying molecular mechanisms has significantly increased. In this context, Genome Wide Association Studies (GWAS) have been widely used to identify novel susceptibility genes in SLE, with more than 60 loci found to be associated with the disease [5], including, for example, alleles of genes located in the human leukocyte antigen (HLA) region [6]. Gene expression signatures have been also largely analyzed across different cohorts, leading to the uncovering of abnormal expression of interferon type I inducible genes in SLE patients, which has been termed the interferon gene signature [7,8]. Recent works have attempted to establish a molecular classification of SLE using omics data [9,10,11]. Banchereau et al. [9] established seven groups of SLE based on gene expression correlation with disease activity. Toro-Dominguez et al. [10] described longitudinal SLE subgroups that differ in clinical features, with two groups that showed high correlation with the percentage of neutrophils and lymphocytes. Finally, an integrative analysis of transcriptome and methylation data stratified different systemic autoimmune diseases, including SLE, into four groups defined by genetic, clinical, serological, and cellular features [11]. Despite all these significant discoveries, we are still at an early stage in the understanding of the mechanistic molecular networks that drive SLE. Besides, different studies have reported that autoimmune disease-associated loci are enriched in transcription factor binding sites and gene regulatory regions [12,13]. These findings are mainly based on the analysis of sequence motifs in SLE-loci or physical interactions with transcription factors (TFs), which do not provide information about TFs activities, the mode of regulation of the TFs, or cell or sample-specific regulatory circuits.

Deciphering TF activity can provide models of regulatory mechanisms to explain the observed changes in gene expression patterns. During the last few years, different studies have shown that TF activity can be inferred from the expression levels of its targeted genes, known as TF-regulon [14]. This approach has been successfully used for the analysis of several types of cancer [15] or to evaluate drug sensitivity [16], among other applications.

In this work, we have systematically analyzed the TFs activity patterns of almost all known human TF-regulons in two gene expression cohorts of SLE patients and control samples. We first estimated the activity of TFs for each sample using DoRothEA [17], a curated target gene database, and analyzed TFs that showed a marked differential activity in SLE with respect to healthy samples. We also explored the stratification of SLE patients based on TFs activity profiles in both SLE cohorts. We obtained consistent results from the analysis of the two independent datasets, finding two main groups of SLE patients based on TFs activity patterns, which were in agreement with previous evidence about SLE stratification [10]. Moreover, we defined the set of TFs with differential activity in SLE samples that provides evidence about the regulatory circuits associated with SLE and disease manifestations.

## 2. Materials and Methods

### 2.1. Data Selection and Preprocessing

We used two independent gene expression datasets, one from adult and another from pediatric SLE patients, as previously described [10]. The pediatric set is composed of 158 SLE patients and 46 healthy controls and the adult dataset comprises 301 SLE patients and 20 healthy samples.

Raw data files were downloaded from Gene Expression Omnibus (GEO) (GSE65391 and GSE121239). For the adult dataset, Affymetrix Cel files were used and background correction and normalization were performed with rma function from affy R package. Normalized values of the pediatric dataset were downloaded from GEO series GSE65391 using the R package GEOquery. Sample processing pipeline is described in the original publication [9]. Array probes were annotated with gene symbols and duplicated genes were merged to their median expression value. SLE samples with SLEDAI greater than 5 were selected first because a SLEDAI score > 5 is associated with a probability of initiating therapy in > 50% of cases [18]. Then, for each patient only the sample with the highest score was used for further analysis. Table 1 shows a summary of the dataset including clinical characteristics that were used in our analysis, such as total white blood cell counts (WBC), erythrocyte sedimentation rate (ESR), levels of C3 and C4 in serum, and percentages of lymphocytes, neutrophils and monocytes. Pediatric data was obtained following the protocols approved by the Institutional Review Boards at the University of Texas Southwestern Medical Center (092010-067) and Baylor University Medical Center (011-200) and informed consent was obtained from adults and the parents or guardians of those younger than 18 years of age according to [9]. Adult data from the SPARE [19] were obtained following the protocol approved by Johns Hopkins University School of Medicine Institutional Review Board. Patients were enrolled from the Hopkins Lupus Cohort after informed consent was obtained and the studies were carried out in accordance with the Helsinki Declaration.

### 2.2. Inferring Transcription Factor Activities

Transcription factor activity from the two independent cohorts was estimated using DoRothEA, a curated database of transcription factor-target gene interactions. The methodology is described in detail in [16,17]. Briefly, the normalized gene expression levels were scaled and recentered and the transcription factor activity was calculated using aREA (analytic rank-based enrichment analysis) algorithm from VIPER R package. Briefly, this algorithm performs an enrichment analysis of the ranked gene expression signature for each TF-regulon, inferring the TF activity of each TF using the expression of its target genes in a sample-to-sample approach. We used the TF regulons from DoRotheA with the highest confidence in order to avoid false positives. The final consensus transcription factor regulons (CTFRs) consisted of 168 TFs and 2602 unique targets. This method allowed us to build a transcription factor activity matrix with TFs in rows and samples in columns and each entry of the matrix representing a normalized enriched score of transcription factor activity in each sample.

### 2.3. Subgroup Identification

Hierarchical clustering analysis (using Euclidean distance and average agglomerative method) and principal component analysis were applied to the TFs activity matrix in order to establish sets of samples with similar TF activity patterns. To define the number of clusters for each dataset we used Calinski and Harabasz index implemented in the R package NbClust.

### 2.4. Differential Activity and Statistical Analysis

The TFs activity matrix was also used to analyze differential activity between SLE and healthy controls in each dataset. We performed this analysis using linear models implemented in limma R package. Transcription factors with False Discovery Rate (FDR) < 0.05 were considered statistically significant for further analysis.

Gene Set Enrichment Analysis (GSEA) was applied with the list ranked by the t value of the differentially expressed genes between SLE and healthy controls and the target genes of each TF in order to obtain the leading-edge subsets for each TF. These subsets are the gene lists that contribute the most to the enrichment score, and they are the most differentially expressed target genes for every TF. Functional enrichment analysis was performed using the Enrichr webtool. In order to identify drugs that target the significant TFs we queried the CLUE database [20]. We downloaded the full drug information from the Repurposing tool and selected those drugs whose targets are significant TFs.

For statistical analysis among clinical variables (SLEDAI, % Neutrophil, % Lymphocyte, % Monocyte, ESR, WBC, C3 and C4) between the SLE groups obtained by subgroup identification the Mann–Whitney U test was used.

## 3. Results

### 3.1. Analysis of TF Activity Revealed Two Main Groups of SLE Patients

SLE is a very heterogeneous disease and there is consistent evidence about differences in global gene expression programs among different SLE patients. In order to evaluate if TFs activity patterns can reveal SLE subgroups, we first performed clustering analysis of SLE samples in both datasets.

**Figure 1:**
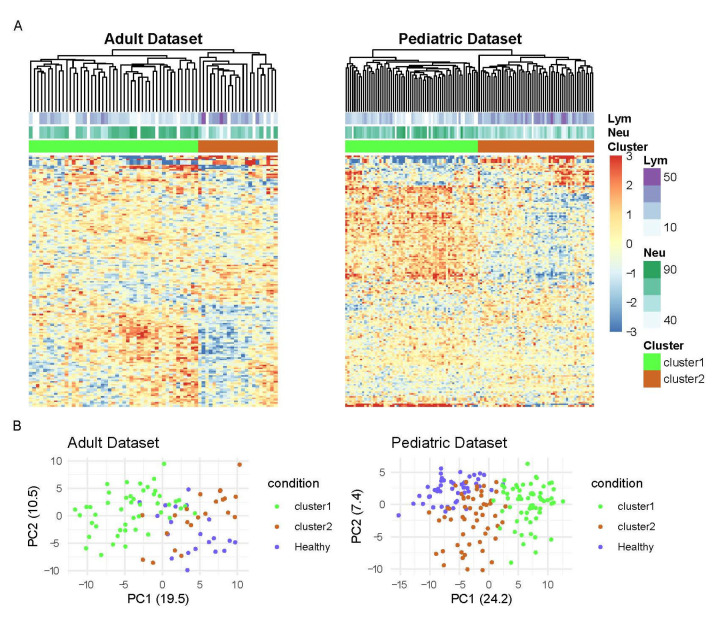
Clustering analysis of transcription factors (TFs) activity profiles in SLE. (**A**) Unsupervised hierarchical clustering of lupus samples based on TFs activity patterns. Top color bars represent percentage of lymphocytes and neutrophils and cluster group. (**B**) Plot showing projection in the two principal components of SLE samples from two clusters together with healthy samples.

**Figure 2:**
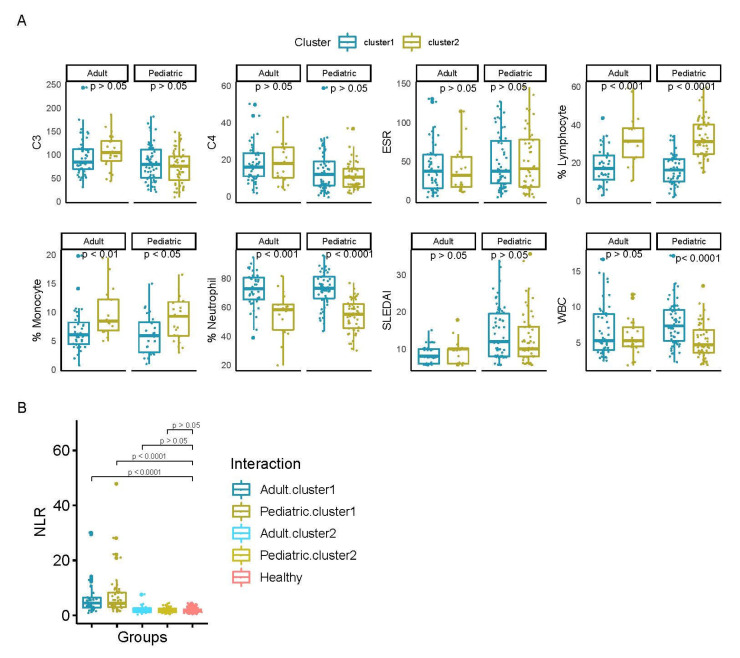
Clinical characterization of SLE clusters. (**A**) Representation of the differences between each SLE cluster in both datasets for each of the clinical variables. Asterisks represent the significance and ‘ns’ the non-significance. (**B**) Depiction of the changes of NLR between each cluster respect to healthy. Numbers represent the significance levels. Mann–Whitney U test was used to compare among groups.

Unsupervised clustering analysis of SLE patients identified two main groups in both datasets (Figure 1A). Cluster 1 in the adult cohort is composed of 47 samples (68%) while cluster 2 included 22 samples (32%). In the pediatric cohort, cluster 1 consisted of 62 samples (53%), whereas cluster 2 is composed of 54 samples (47%). The analysis of clinical variables of samples from each cluster revealed a significant difference in the proportion of samples that were enriched in lymphocyte (Adults: *p* =1.76 × 10^−4^ and Pediatric: *p* < 0.0001 1.33 × 10^−12^) and neutrophil percentages (Adults: *p* = 1.96 × 10^−4^ and Pediatric: *p* < 0.0001) in each group (Figure 2A). While cluster 1 was enriched in samples with higher neutrophil percentage, cluster 2 contained the most samples with a higher percentage of lymphocytes. We also analyzed the distribution of the other cellular proportions using cibersort, a deconvolution algorithm [21] and although differences in other cell types were observed in one of the cohorts, we did not find significant differences across clusters that were consistent in both datasets (See Figure S1). These findings were consistent in both independent datasets, and they were in agreement with our previous observations reported in [10] where we described a stratification of SLE patients into three main groups, mainly associated with differences in the percentages of these cell populations when disease activity increases.

Interestingly, principal component analysis of the SLE and healthy samples revealed that healthy individuals were grouped together with the subset of SLE samples enriched in lymphocytes (Figure 1B). Although information about the cell populations of healthy samples was not available, we used a previously published dataset that evaluated the normal value of the neutrophil-to-lymphocyte ratio (NLR) from 413 healthy samples [22] to have a reference of the NLR values for healthy samples. Therefore, we compared the NLR values from each cluster with the NLR values from the healthy set. In both datasets, the NLR values of SLE samples in cluster 1 were significantly higher than in the healthy samples (Adults: *p* < 0.0001 and Pediatric: *p* < 0.0001). On the other hand, the cluster 2 samples showed similar NLR values to the healthy individuals (Adults: *p* = 0.6441 and Pediatric: *p* = 0.8143) (Figure 2B).

Taken together, these results show that activity patterns of TF-regulons can distinguish between two main groups of SLE patients that are characterized by differences in neutrophil/lymphocyte proportions. In addition, there is clear evidence of the shared transcription activity patterns between SLE patients enriched in lymphocyte proportions and the healthy controls.

### 3.2. TFs with Differential Activity in SLE and Healthy Samples

In order to establish the set of TFs operating in SLE we first performed a differential activity analysis of all SLE against healthy samples. We found a set of 49 TFs that showed significant differential activity between SLE and healthy samples in both datasets (Figure S2). Nevertheless, there was intragroup heterogeneity in the activity value of these TFs. For example, activity patterns for MYC, RFX5, RFXAP and RFXANK were clearly different across the two clusters of SLE samples described previously. Interestingly, the expression level of most of the target genes of these TFs varied within different cell types collected by Expression Atlas (Figure S2B). For example, the expression of HLA genes, regulated by RFXANK, RFXAP and RFX5, was much higher in B cell types and dendritic with respect to the rest. On the other hand, there are some genes, such as BBC3, CXCL2, ICAM1, PTEN, EGR3, HBA2 or IMPA2, regulated by MYC, whose expression was higher in neutrophil cell types. This fact might reflect the differences in cell proportions found in the two SLE clusters.

**Figure 3:**
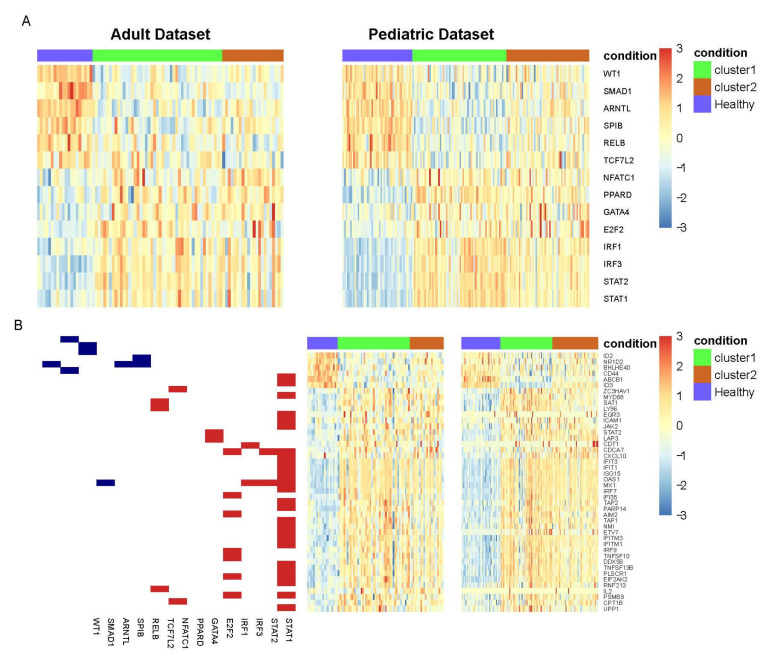
Robust Differential Activity Analysis across SLE and healthy samples. (**A**) Heatmaps show normalized activity values of 14 transcription factors that were found consistently significant across SLE patient and healthy samples in the two independent cohorts (**B**) Gene expression values of leading-edge subsets genes. On the left-hand side there is a graph showing target genes for each TF. Blue and red colors indicate which TFs had lower activity and higher activity in SLE with respect to healthy samples, respectively. Heatmaps on the right-hand side show gene expression values of the corresponding genes (found in the leading-edge subset). Top bars indicate the cluster assignation for each SLE patient as well as healthy samples.

Therefore, we compared each SLE cluster independently against healthy samples in order to identify TFs that consistently showed differential activity with respect to healthy individuals. This analysis revealed a set of 96 and 60 TFs in cluster 1 and cluster 2, respectively, for the adult dataset, and 135 and 57 TFs in cluster 1 and 2, respectively, for the pediatric set. Sixty-nine TFs in cluster 1 overlapped in both sets (Figure S3A) while 21 TFs in cluster 2 had significant differential activity between healthy samples and both datasets (Figure S3B). From these results, 14 TFs were consistently activated or repressed across all SLE samples, regardless of SLE subgroup (Figure 3A). Specifically, SMAD1, ARNTL, WT1, RELB, SPIB and TCF7L2 had lower activity in SLE while GATA4, NFATC1, E2F2, PPARD, IRF3, STAT2, IRF1, STAT1 were identified as TFs with greater activity.

We next evaluated which was the set of target genes whose expression contributed more to the activity signal of the 14 TFs defined in the analysis. To this end, a GSEA was carried out to identify the leading-edge subset of target genes most differentially expressed between SLE clusters and healthy samples for each TF. This analysis yielded a total of 44 target genes (Figure 3B). In this figure it is shown that transcription factors with lower activity in SLE with respect to the healthy controls were involved in the regulation of the underexpressed genes, whilst the overexpressed genes were regulated mainly by the transcription factors with higher activity. Not unexpectedly, most overexpressed genes were involved in interferon signaling pathways and susceptibility to viral infection, which are mainly the targets of STAT1. On the other hand, the few underexpressed genes are related to photoperiodism and circadian clock activity. These biological processes have been previously reported in SLE and other autoimmune diseases but the association has not been well-studied [23,24]. To complete the analysis, we evaluated drugs that can potentially target the set of significant TFs obtained using information from the CLUE database. To this end, we defined the set of drugs that target directly these TFs (Table 2). From the 15 drugs obtained, PPARD is the TF that is associated with 12 of these drugs, most of them related to Perixosome proliferator receptor (PPAR), which has been described to be associated with Lupus-like autoimmunity development (see discussion). In addition, we found drugs that target NFATC1, IRF3 and STAT1. Interestingly, all the discovered drugs’ target TFs had higher activity in SLE compared to healthy controls.

## 4. Discussion

Systemic lupus erythematosus is a complex and heterogeneous disease with limited diagnosis and treatment options. Despite research efforts and clinical trials that have been conducted to establish new treatments, only belimumab, a monoclonal antibody against B cell-activating factor (BAFF), has been approved in the last few decades [25]. Therefore, there is an urgent need to decipher the main biological mechanisms underlying SLE to expand our knowledge about the disease and define new biomarkers, classification schemes and treatment options. In the last few years, a large number of SLE biomarkers have been described by GWAS and whole transcriptomics analysis. Interestingly, the positional analysis of these SLE associated loci has revealed that most of them are located in regulatory regions, but there is a lack of a global analysis of TFs activity patterns associated with SLE disease.

In this work we have inferred the TF activities for most human TFs using gene expression levels of SLE patients with active status of the disease in two different cohorts. First, the unsupervised clustering analysis of the activity matrix revealed a two-cluster structure in both datasets, characterized mainly by differences in the neutrophil and lymphocyte proportions. Our molecular classification and clinical characterization are coherent with previous works that stratified SLE or systemic autoimmune diseases in which different proportions of neutrophils and lymphocytes were associated with different groups [9,10,11]. In this context, there is recent evidence about the potential role of neutrophils’ and lymphocytes’ proportions as potential markers to stratify SLE patients into clinically separate groups [26,27]. Indeed, the potential of the neutrophil-lymphocyte ratio (NLR) as a cheap and effective biomarker of the activity or response to treatment in autoimmune pathologies is being analyzed by different groups [28,29,30] and it has also been described that alterations in the balance of neutrophils, monocytes and lymphocytes explain resistance to treatments in patients with rheumatoid arthritis [31], although the molecular mechanisms are not totally characterized

After comparing TF activities from SLE and healthy samples, we defined 49 TFs with significant differential activity. A detailed analysis revealed that some of them could be biased by the heterogeneity of cell population in SLE samples, which is remarkable for the TFs MYC, RFX5, RFXAP and RFXANK. RFX5, RFXANK and RFXAP, that act as enhancers of the gene expression of the Major Histocompatibility Class II genes (MHC II) [32]. These are expressed in professional antigen-presenting cells (APCs) [33] such as monocytes, B cells and dendritic cells. This could explain the low activity of these TFs in cluster 1, representing neutrophils. On the other hand, MYC regulates a large set of genes, but the presence of ribosomal genes such as RPS15, RPL19, RPS19, RPS6, RPL3, RPL22, RPL6, RPL32, RPL27A, RPL23 and RPS16 stands out. The expression of these genes in different types of blood cells is very heterogeneous [34].

Due to the observed heterogeneity in TF activities of SLE patients, we decided to compare TF activities of each cluster with healthy samples in order to obtain consistent and unbiased regulation patterns across all SLE patients. From these analyses we established 14 TFs that were consistently activated or repressed in SLE.

Those with greater activity in SLE were STAT1, STAT2, IRF1, IRF3, NFATC1, PPARD, E2F2 and GATA4. As expected, this set contains transcription factors that are well-known in the context of SLE pathogenesis including STAT1, STAT2, IRF1 and IRF3 which are activators of interferon genes [35,36]. The analysis of drug–TFs association revealed that STAT1 is the target of CKD-712, which is an inhibitor of NF-κB pathway, a proinflammatory mediator [37]. On the other hand, a SYK inhibitor acts on IRF3. This mechanism has been shown to be effective in rheumatoid arthritis and lupus-prone MRL/lpr mice [38]. In fact, the overexpression of Syk in healthy T cells leads to a SLE-like T cell phenotype, suggesting that the inhibition of Syk gives the opposite effect. Syk has been proposed as a therapeutic target [39].

NFATC1 is overexpressed in lupus-prone MRL/lpr mice activating the calcium/NF-AT pathway [40]. Moreover, this TF is the target of a preclinical calcineurin inhibitor [41], the inhibitory mechanism through which cyclosporine and tracolimus exert their effects when used in SLE patients [42].

Mice deficient in Ppard develop Lupus-like autoimmunity with increased production of autoantibodies and abnormal apoptotic cell clearance [43]. There are some drugs whose target is PPARD (Table 2). One of the most noteworthy drugs that target PPARD, tretinoin, is related to the mechanism of action (MoA) retinoid receptor agonist. The improvement of the inflammatory symptoms of SLE has been reported with retinoic acid treatment in murine models and human disease [44]. SLE amelioration could be achieved via retinoic acid treatment through three mechanisms [45]. One of these is by reversing microbial dysbiosis [46]; secondly, by inhibiting the activity of Pin-1, which activates the TLR-7/TLR-9/IRAK-1/IRF-7 signal that contributes to SLE phenotype [47]; and, thirdly, by reestablishing the vitamin A levels in SLE patients, which improves the T helper 17 (Th17) and regulatory T cell (Treg) balance [44]. Additionally, PPARD is a target of sulindac, which is a cyclooxygenase inhibitor launched in clinical trials of rheumatoid arthritis or spondylitis.

The rest of the significant TFs had lower activity in SLE than in controls: SMAD1, ARNTL, WT1, RELB, SPIB and TCF7L2. SMAD1, along with other genes involved in the BMP/Smad signaling pathway, is repressed through NF-κB signaling pathway in bone marrow-derived mesenchymal stem cells (BMMSCs) from SLE patients [48]. On the other hand, RELB is a subunit of NF-κB and takes part in the development of dendritic cells [49]. In the murine lupus model, Relb-modified dendritic cells decreased the interferon-γ expression [50]. ARNTL is a TF that forms a core component of the circadian clock. This system regulates the gene expression of genes involved in several biological processes according to circadian rhythms. As we have described previously, there are studies that correlated circadian clock dysregulation and SLE pathogenesis [23,24].

Lupus nephritis is one of the most severe manifestations of SLE, characterized by the inflammation of the kidneys and the loss of podocytes [51]. WT1 is a well-known podocyte marker [52] and in murine model its expression is decreased [53].

SPIB belongs to the ETS family of TFs and promotes the development of plasmacytoid dendritic cells (pDC), the major producers of type I interferon and is involved in the development of germinal center B cells [54]. However, SPIB has been shown to be underexpressed in the B cells of SLE patients [55]. SPIB, as well as E2F2, GATA4 and TCF7L2 have been associated with other autoimmune diseases through differential gene expression and genetic polymorphisms, respectively [56,57,58].

Although many of the TFs associated with SLE patients have been previously described, in this work we described the stratification of SLE patients into two subgroups based on global TF activity profiles, which are characterized by differences in the neutrophil and lymphocyte proportions. In addition, we identified 14 significant and robust TFs across SLE patients. These results reveal regulation mechanisms regarding SLE heterogeneity, which might be possible therapeutic targets. The groups observed here are consistent with previous findings [10] and can link molecular heterogeneity to clinical manifestations or response to therapies, providing opportunities for novel therapeutic developments or better disease diagnosis.

## Figures and Tables

**Table 1:** Clinical characterization of Systemic Lupus Erythematosus (SLE) patients.

	Adult	Pediatric
Gender	67 female and 2 male	102 female and 14 male
SLEDAI	8.493 ± 2.5	13.371 ± 6.6
% Neutrophil	67.289 ± 15.4	63.963 ± 14.8
% Lymphocyte	22.641 ± 12.9	24.808 ± 12.4
% Monocyte	7.68 ± 4.0	7.415 ± 3.8
C3 (mg/dL)	99.667 ± 39.0	78.645 ± 37.5
C4 (mg/dL)	17.87 ± 10.2	12.495 ± 9.5
WBC (K/cu mm)	6.396 ± 3.3	6.507 ± 2.9
ESR (mm/h)	41.765 ± 31.2	50.038 ± 37.0
Proteinuria	20 patients with proteinuria and 49 without	69 patients with proteinuria and 47 without
Pyuria	11 patients with pyuria and 58 without	41 patients with pyuria and 75 without

**Table 2:** Information about significant TFs that are drugs-targets from the CLUE database.

Drug	Mechanisms of Action (MoA)	TF Target	Indication	Phase
Bezafibrate	PPAR receptor agonist	PPARD	Cholesterol	Launched
DG-172	PPAR receptor inverse agonist	PPARD		Preclinical
Elafibranor	PPAR receptor agonist	PPARD		Phase 3
FH-535	PPAR receptor antagonist, WNT signaling inhibitor	PPARD		Preclinical
GSK-0660	PPAR receptor antagonist	PPARD		Preclinical
GSK3787	PPAR receptor antagonist	PPARD		Preclinical
GW-0742	PPAR receptor agonist	PPARD		Preclinical
GW-501516	PPAR receptor agonist	PPARD		Phase 2
Icosapent	Platelet aggregation inhibitor	PPARD	Hypertriglyceridemia	Launched
L-165041	PPAR receptor agonist	PPARD		Preclinical
Sulindac	Cyclooxygenase inhibitor	PPARD	Osteoarthritis, rheumatoid arthritis, ankylosing spondylitis	Launched
Tretinoin	Retinoid receptor agonist, retinoid receptor ligand	PPARD	Leukemia	Launched
INCA-6	Calcineurin inhibitor	NFATC1		Preclinical
piceatannol	SYK inhibitor	IRF3		Preclinical
CKD-712	NFkB pathway inhibitor	STAT1		Phase 1

## Supplementary Materials

The following are available online at https://www.mdpi.com/article/10.3390/life11040299/s1, Figure S1. Analysis of cellular proportions imputed with Cibersort in SLE clusters. Figure S2. Differential analysis between SLE and healthy samples. Figure S3. Analysis between SLE clusters and healthy samples.
