# Supplementary material for: Transcription Factor Activity Inference in Systemic Lupus Erythematosus
Source: Life (Basel). 2021 Apr 1;11(4):299. doi: 10.3390/life11040299 (PMC8065841; doi:10.3390/life11040299)
Supplement: Supplementary file 1 [file life-11-00299-s001.zip › Supplementary_Figure_2.pdf]

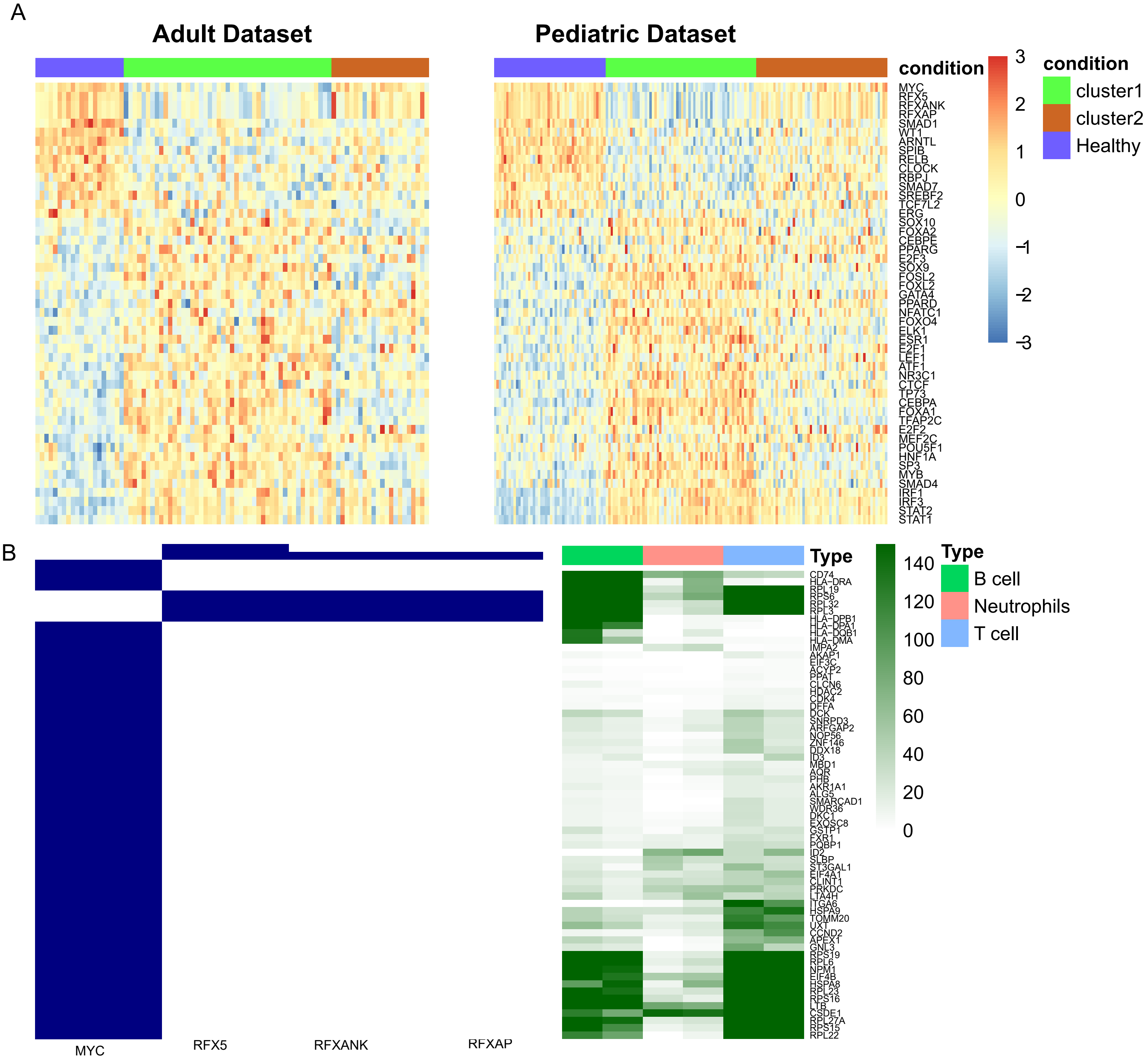
A
Adult Dataset
Pediatric Dataset
condition
MYC
RFX5
RFXANK
RFXAP
SMAD1
WT1
ARNTL
SPIB
RELB
CLOCK
RBPJ
SMAD7
SREBF2
TCF7L2
ERG
SOX10
FOXA2
CEBPE
PPARG
E2F3
SOX9
FOSL2
FOXL2
GATA4
PPARD
NFATC1
FOXO4
ELK1
ESR1
E2F1
LEF1
ATF1
NR3C1
CTCF
TP73
CEBPA
FOXA1
TFAP2C
E2F2
MEF2C
POU5F1
HNF1A
SP3
MYB
SMAD4
IRF1
IRF3
STAT2
STAT1
3
2
1
0
−1
−2
−3
condition
cluster1
cluster2
Healthy
B
MYC
RFX5
RFXANK
RFXAP
Type
CD74
HLA−DRA
RPL19
RPS6
RPL32
RPL3
HLA−DPB1
HLA−DPA1
HLA−DQB1
HLA−DMA
IMPA2
AKAP1
EIF3C
ACYP2
PPAT
CLCN6
HDAC2
CDK4
DFFA
DCK
SNRPD3
ARFGAP2
NOP56
ZNF146
DDX18
ID3
MBD1
AQR
PHB
AKR1A1
ALG5
SMARCAD1
WDR36
DKC1
EXOSC8
GSTP1
FXR1
PQBP1
ID2
SLBP
ST3GAL1
EIF4A1
CLINT1
PRKDC
LTA4H
ITGA6
HSPA9
TOMM20
UXT
CCND2
APEX1
GNL3
RPS19
RPL6
NPM1
EIF4B
HSPA8
RPL23
RPS16
LTB
CSDE1
RPL27A
RPS15
RPL22
140
120
100
80
60
40
20
0
Type
B cell
Neutrophils
T cell
