# Supplementary figures and images for: Transcription Factor Activity Inference in Systemic Lupus Erythematosus
Source: Life (Basel). 2021 Apr 1;11(4):299. doi: 10.3390/life11040299 (PMC8065841; doi:10.3390/life11040299)

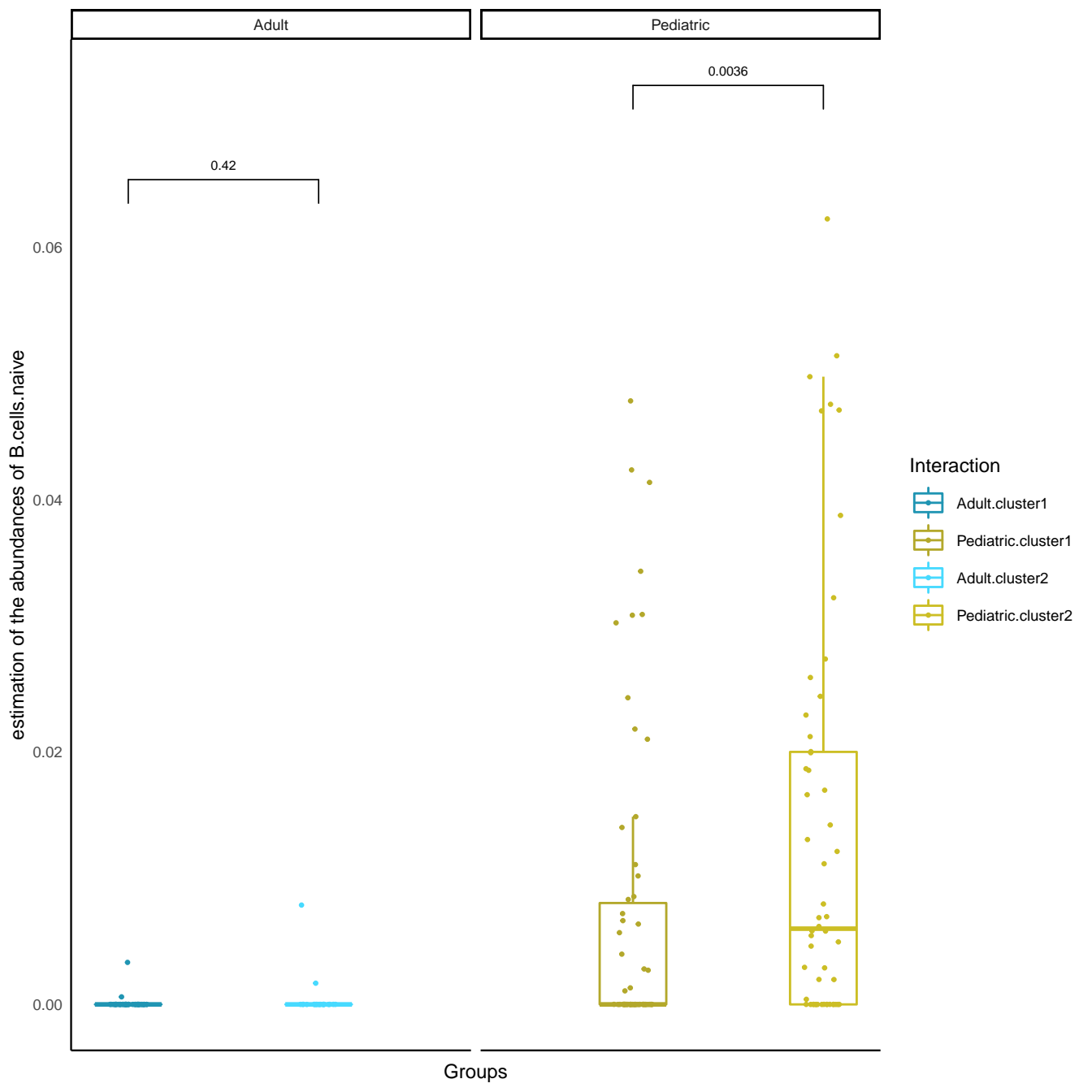

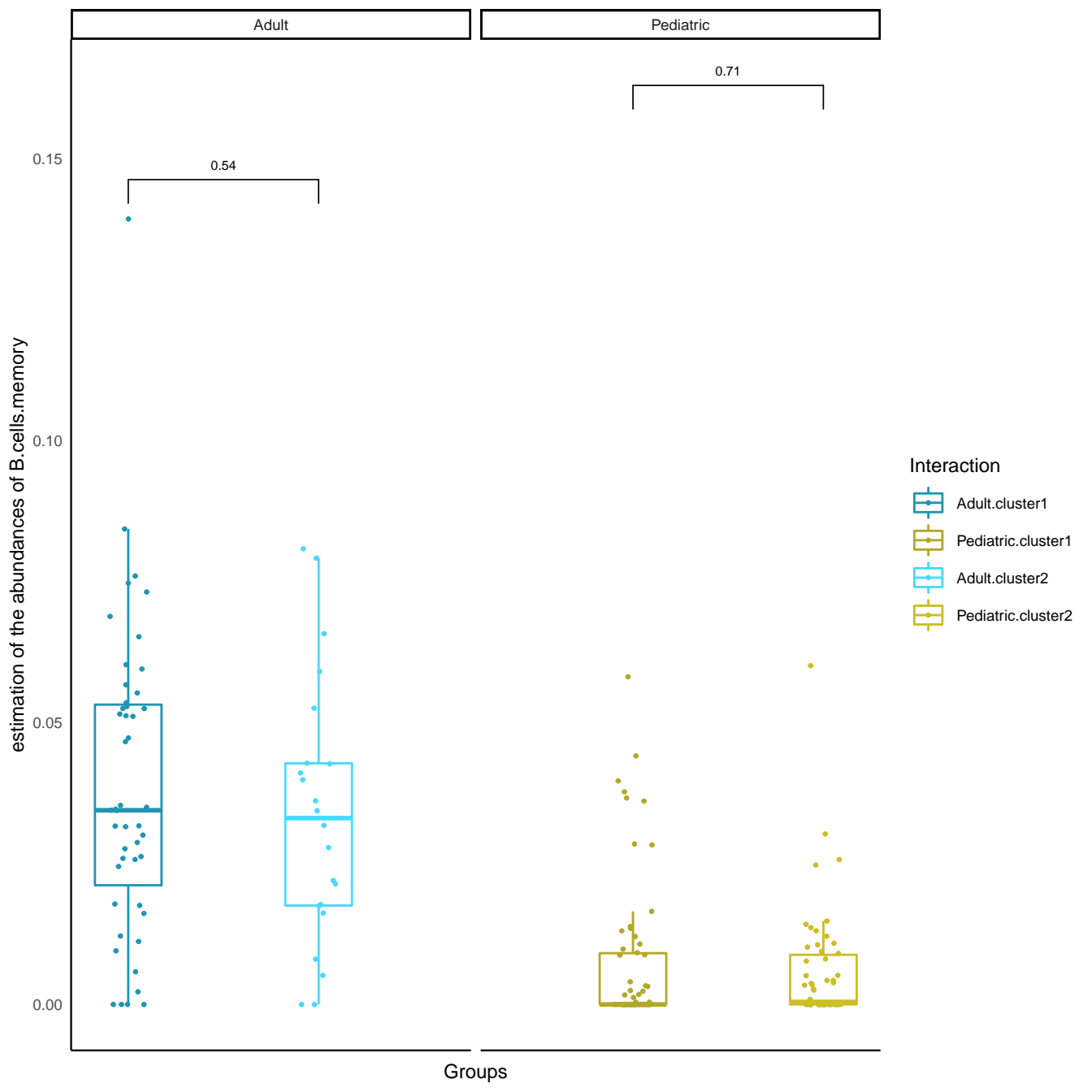

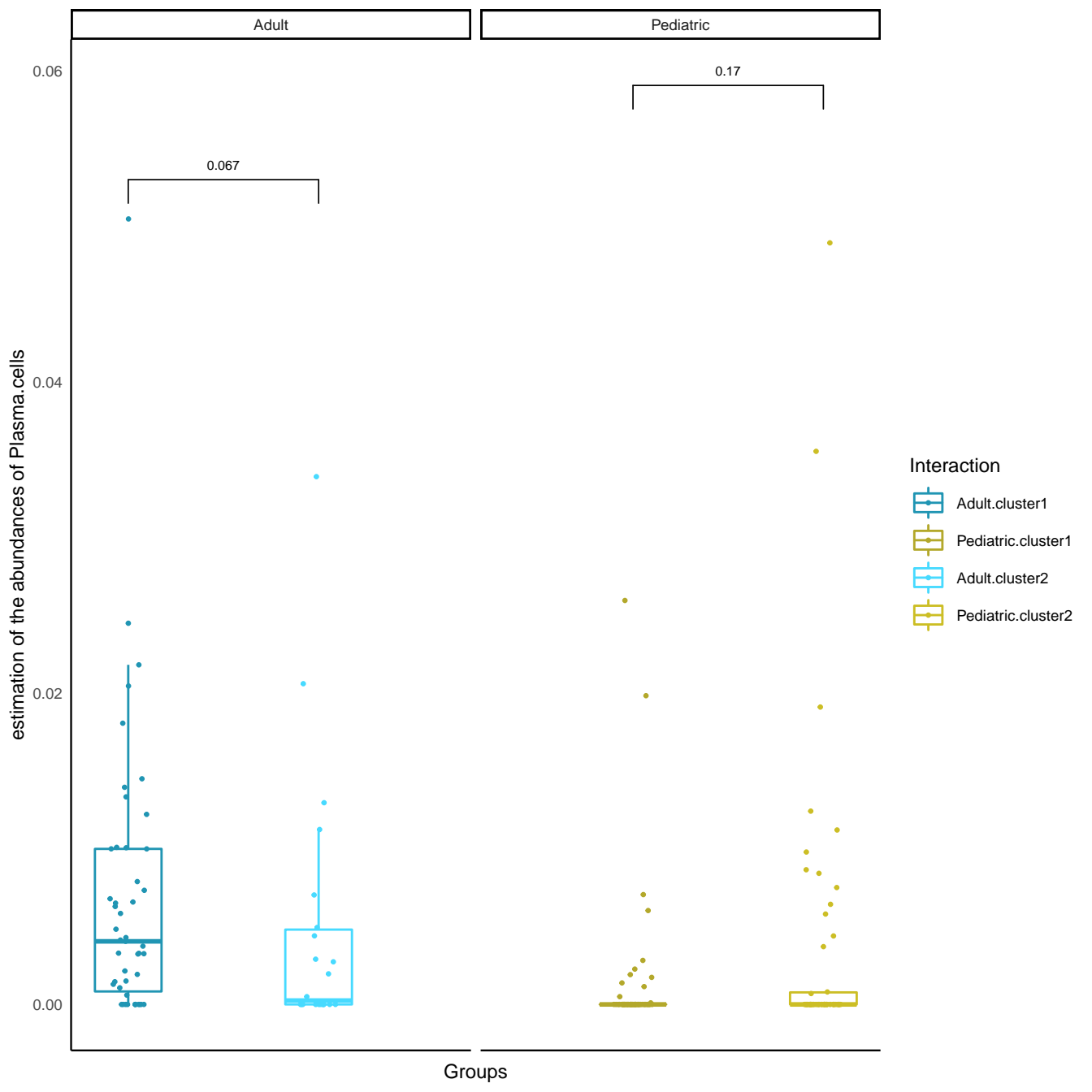

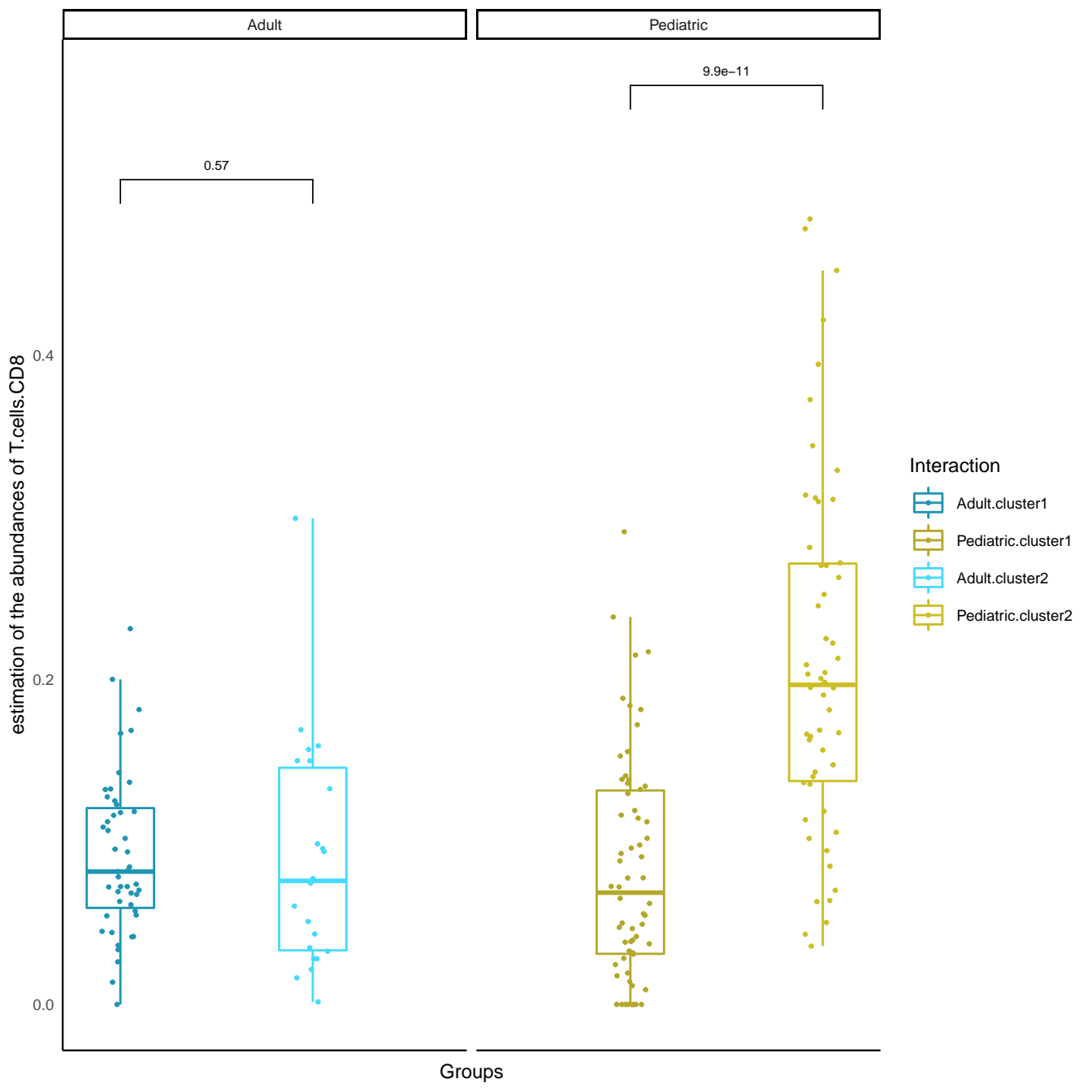

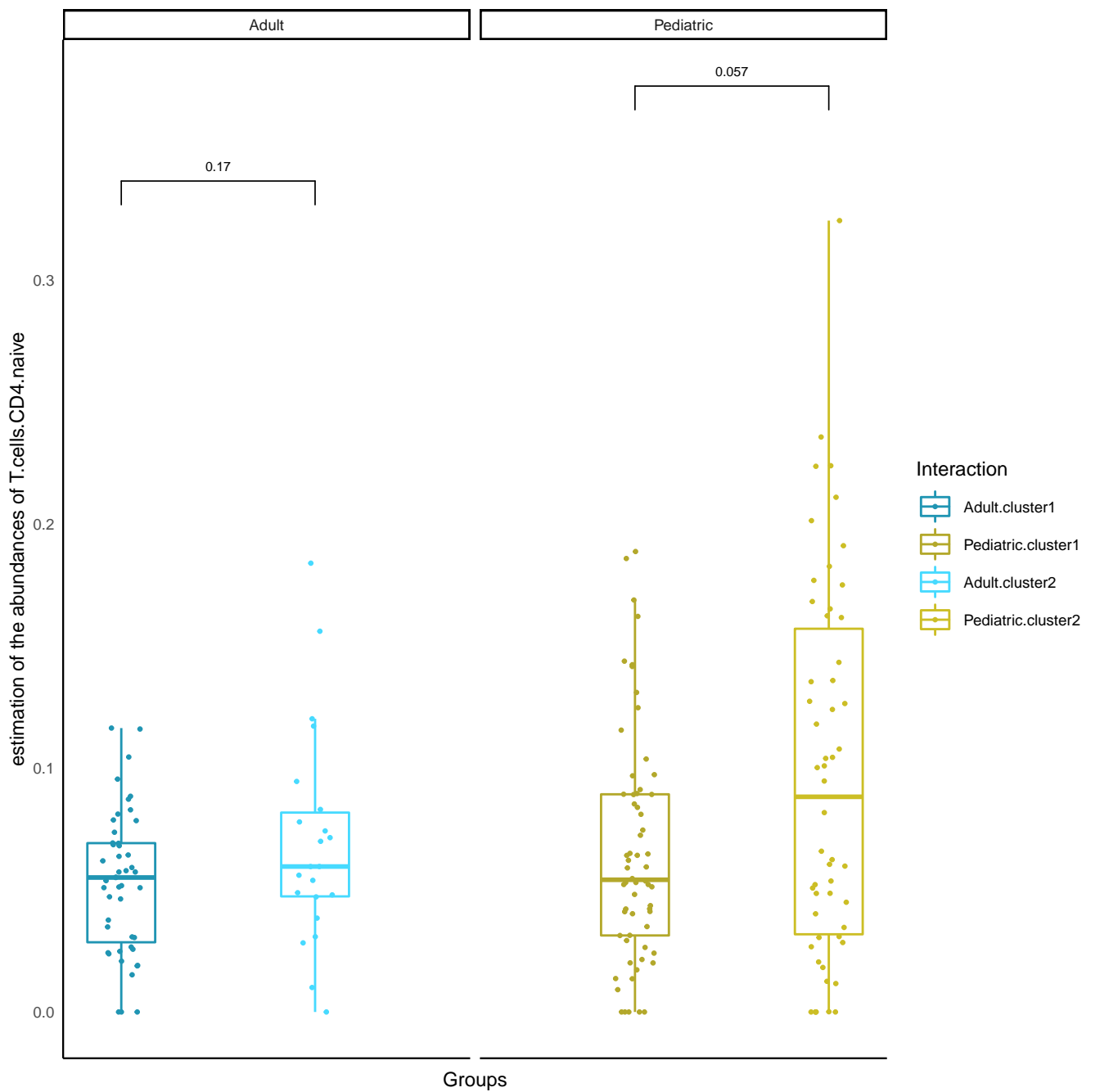

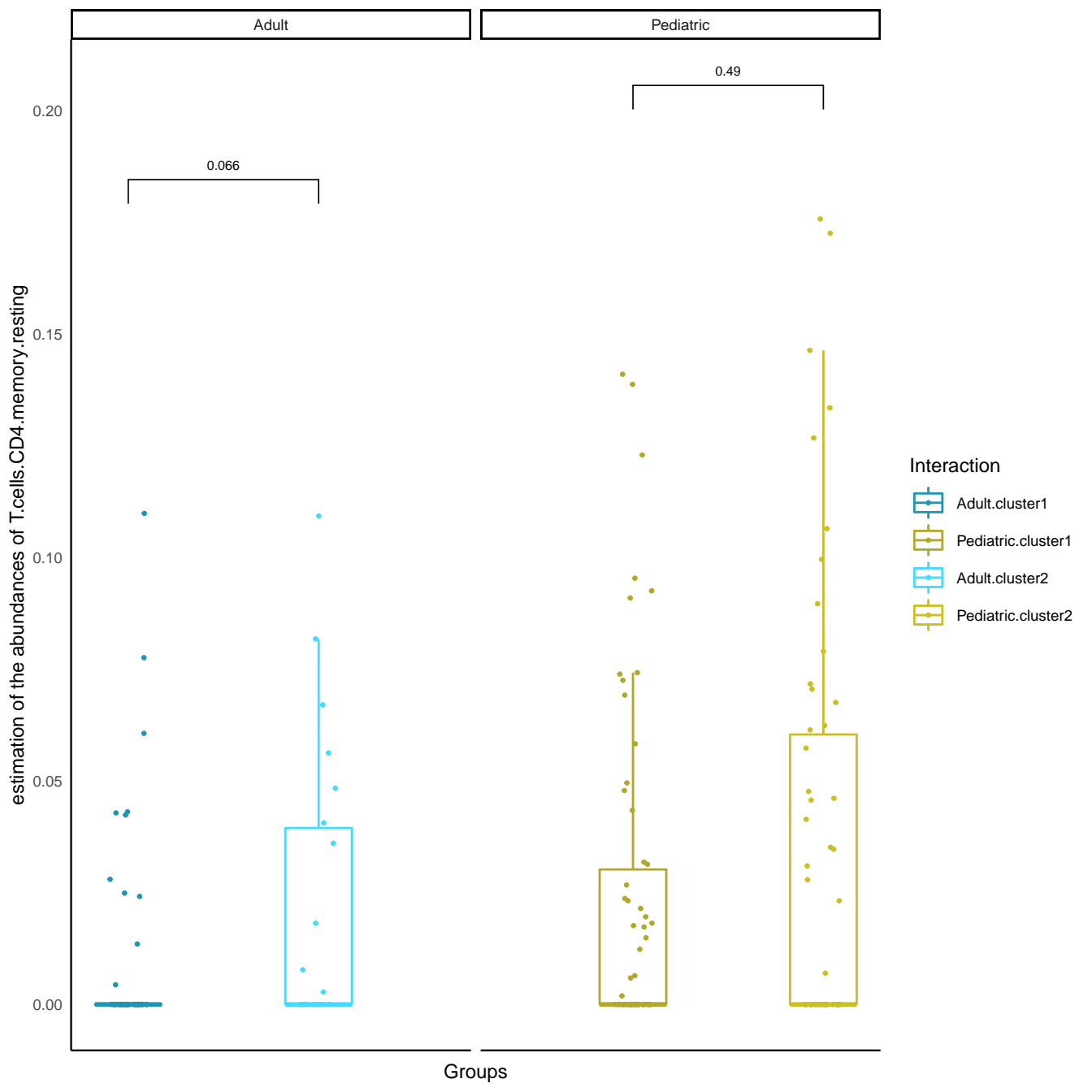

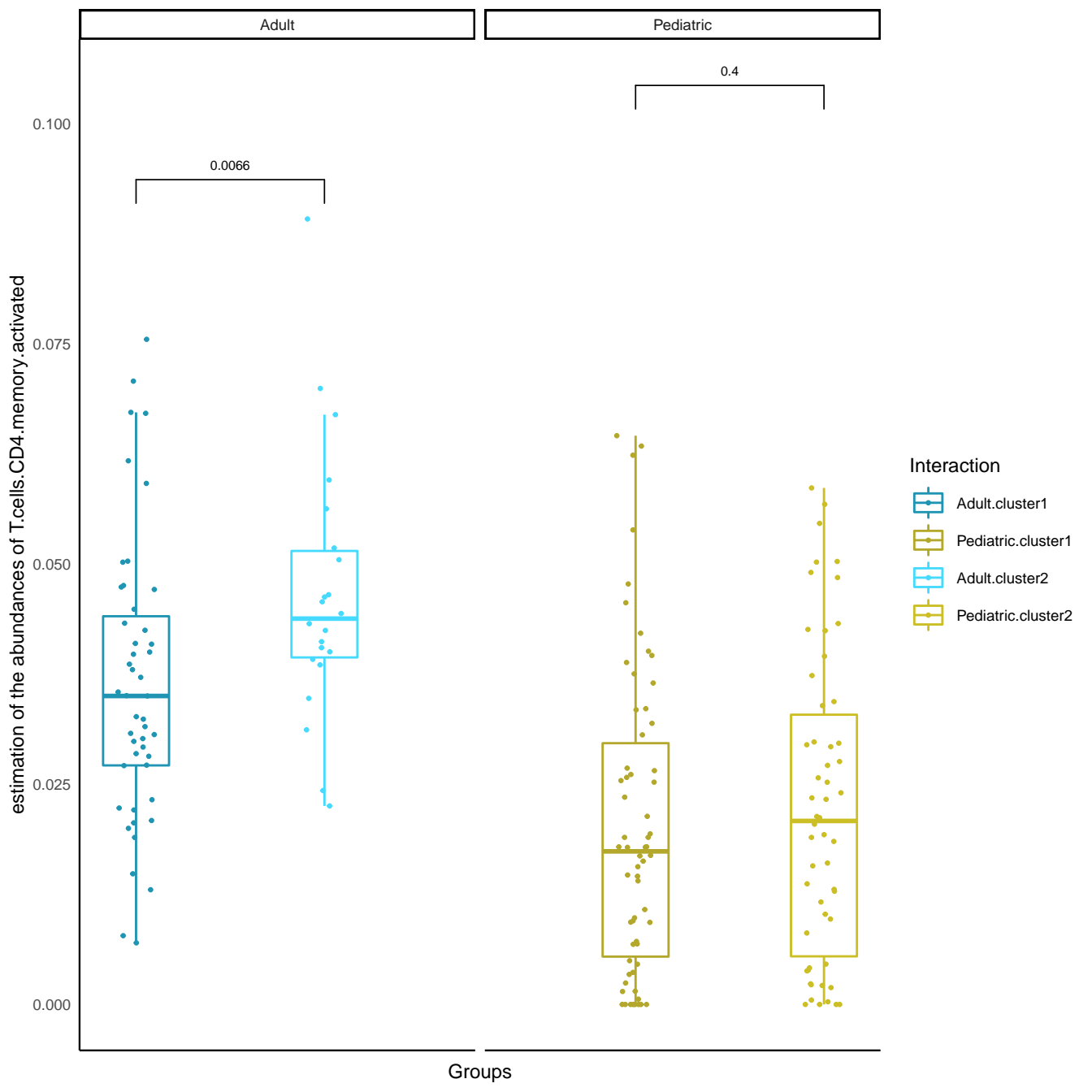

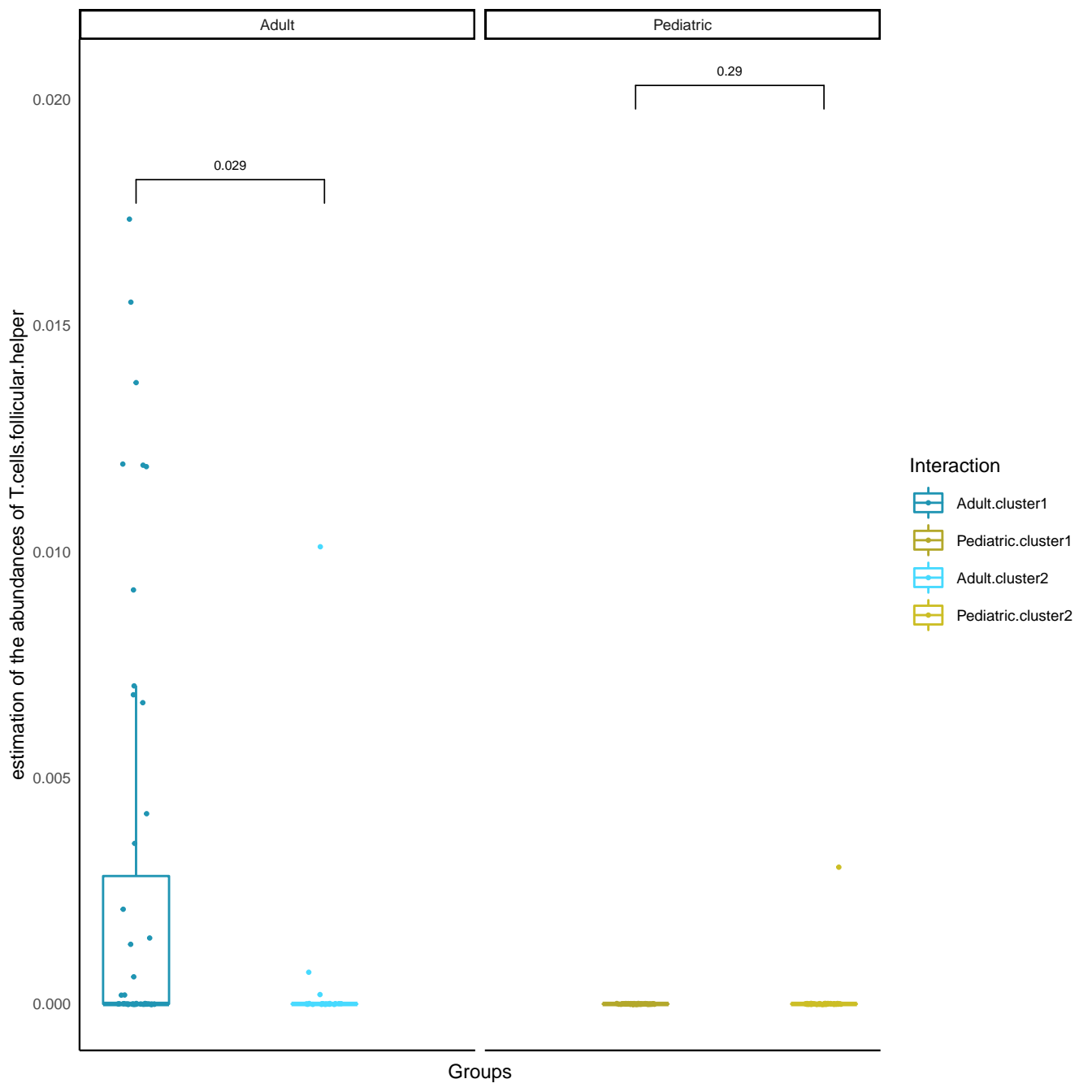

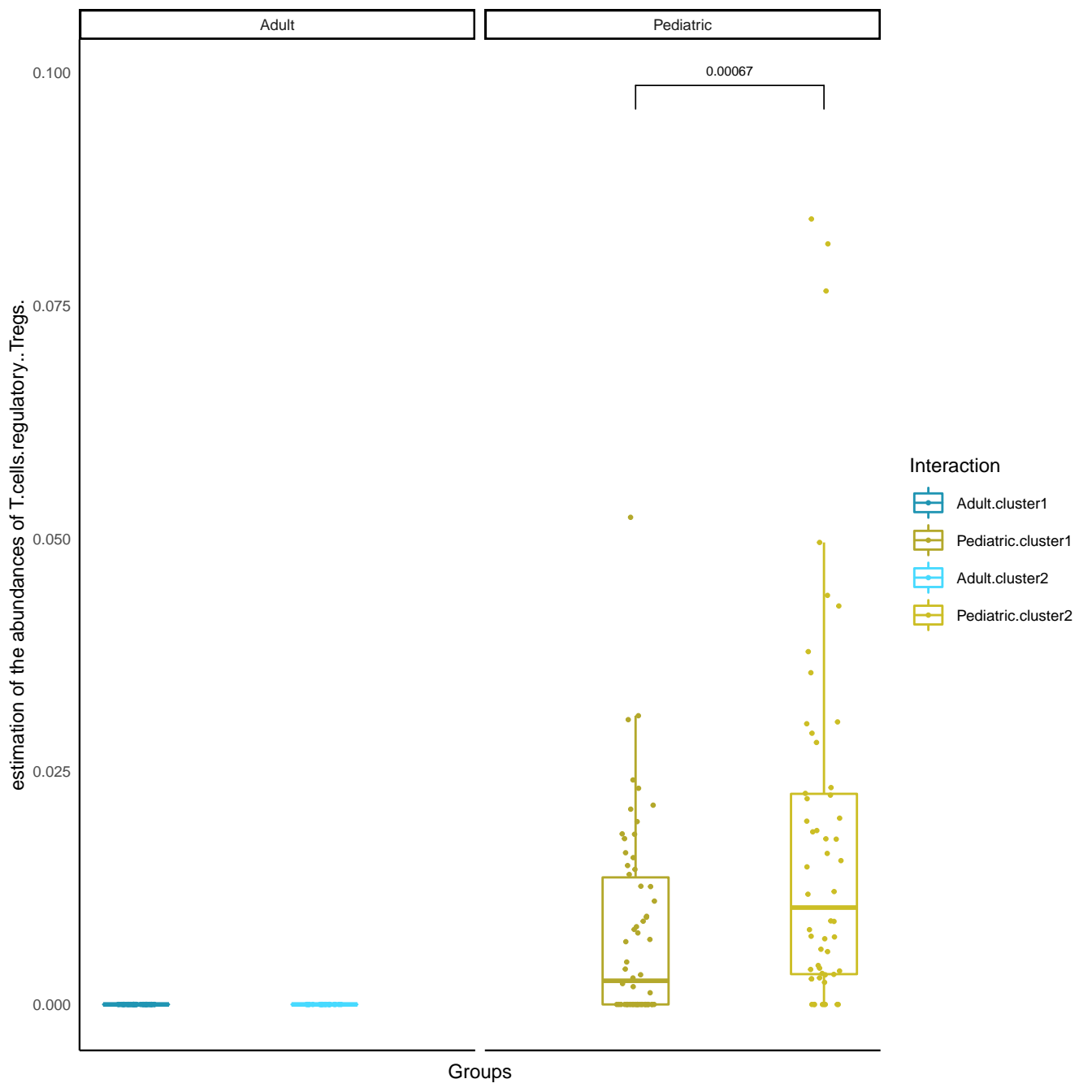

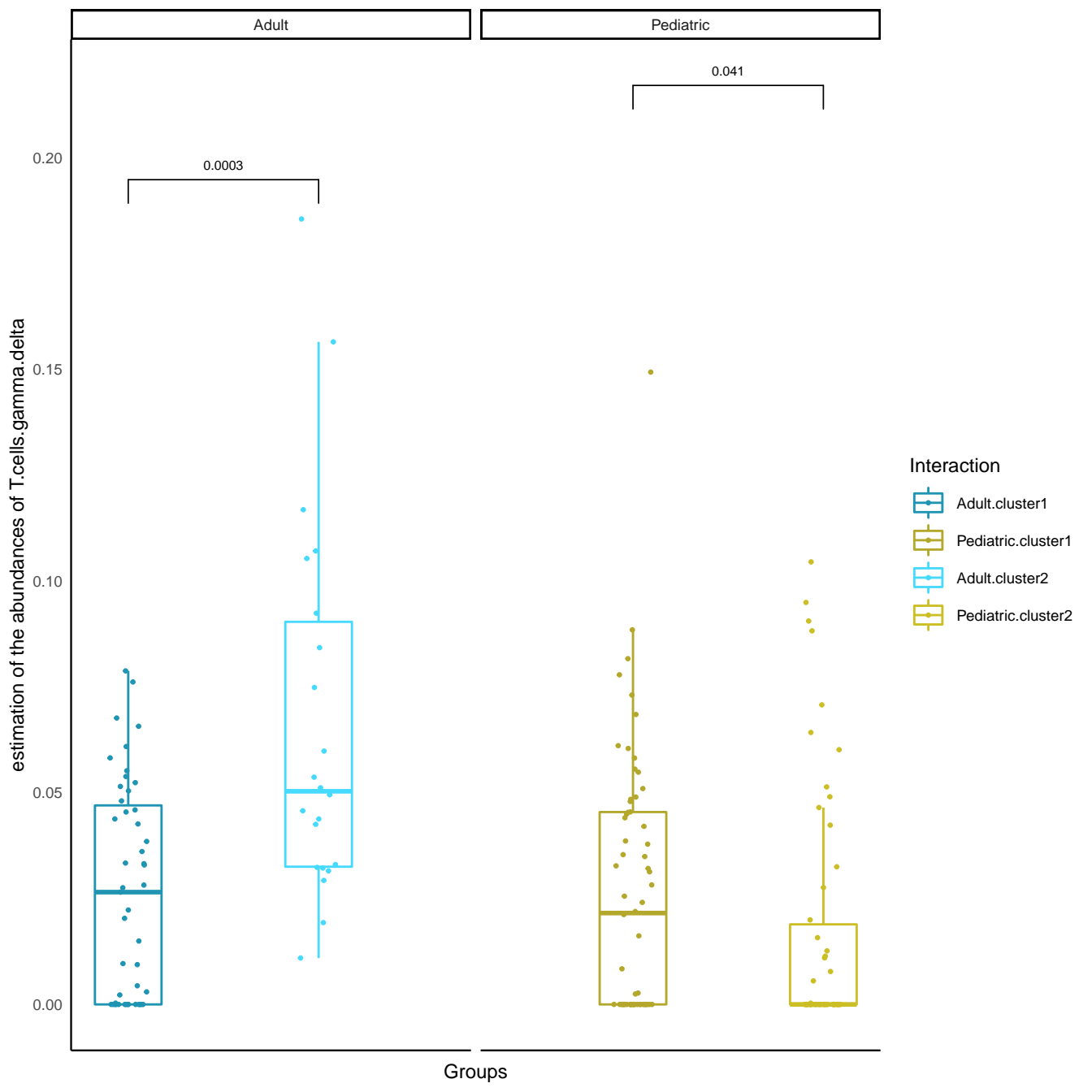

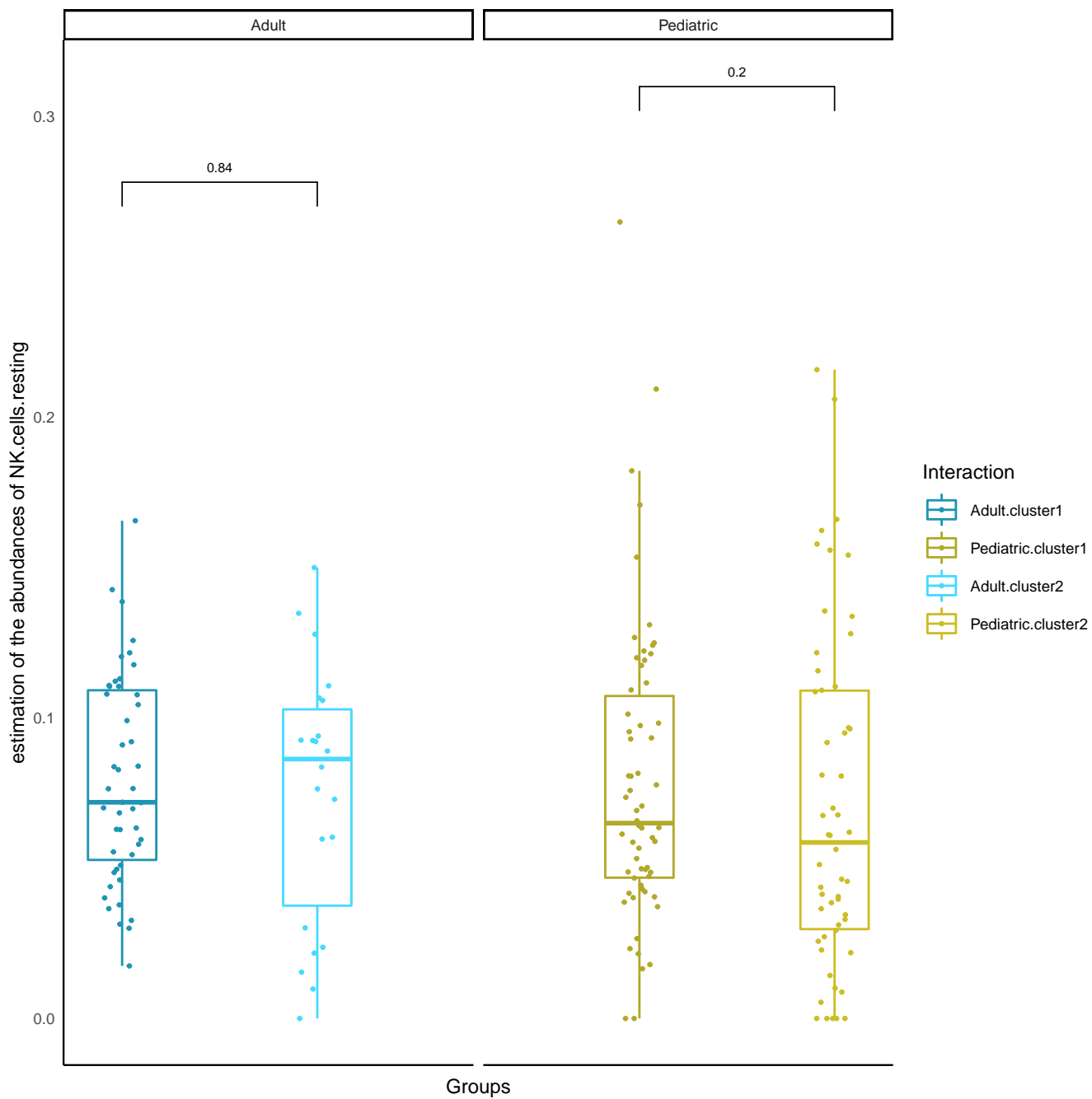

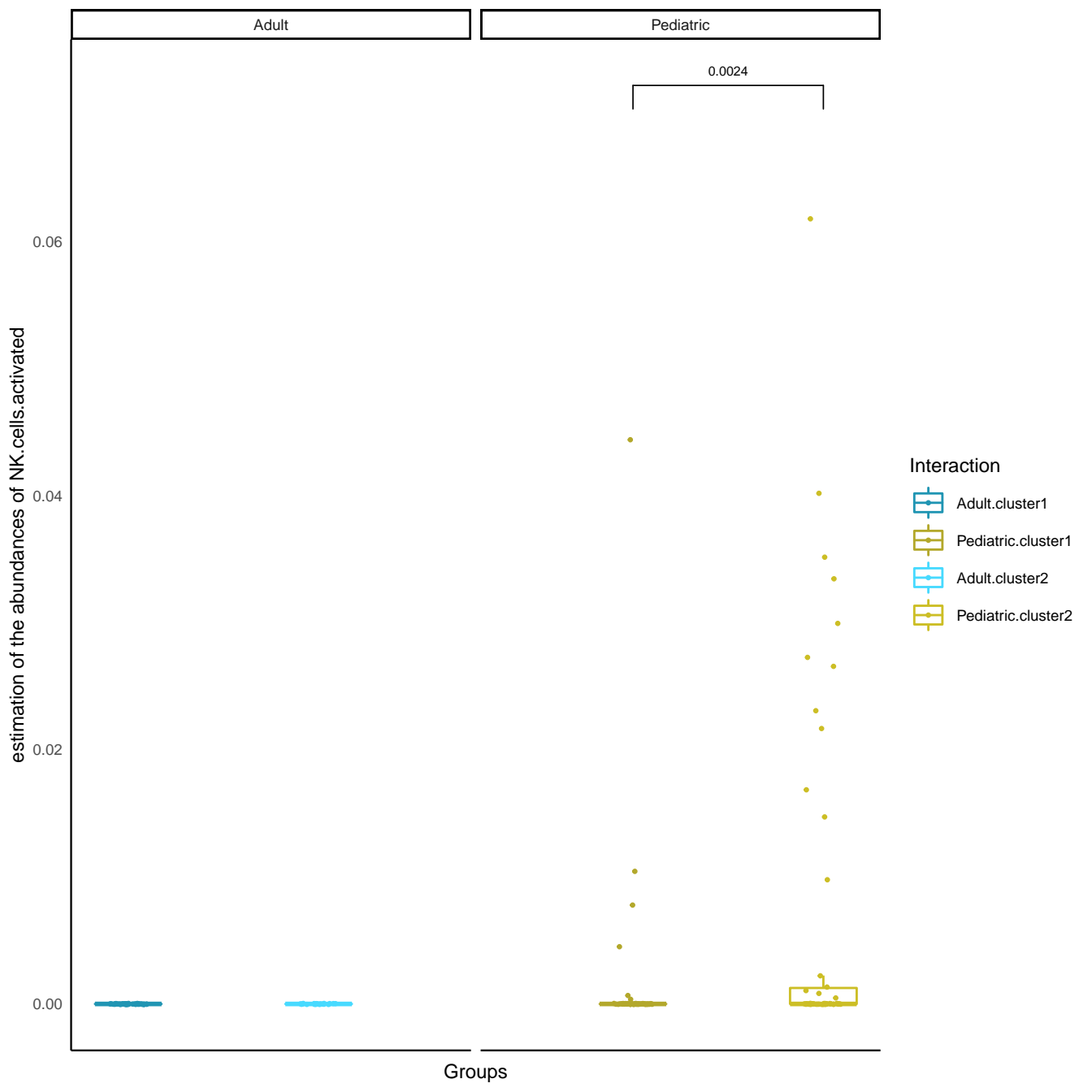

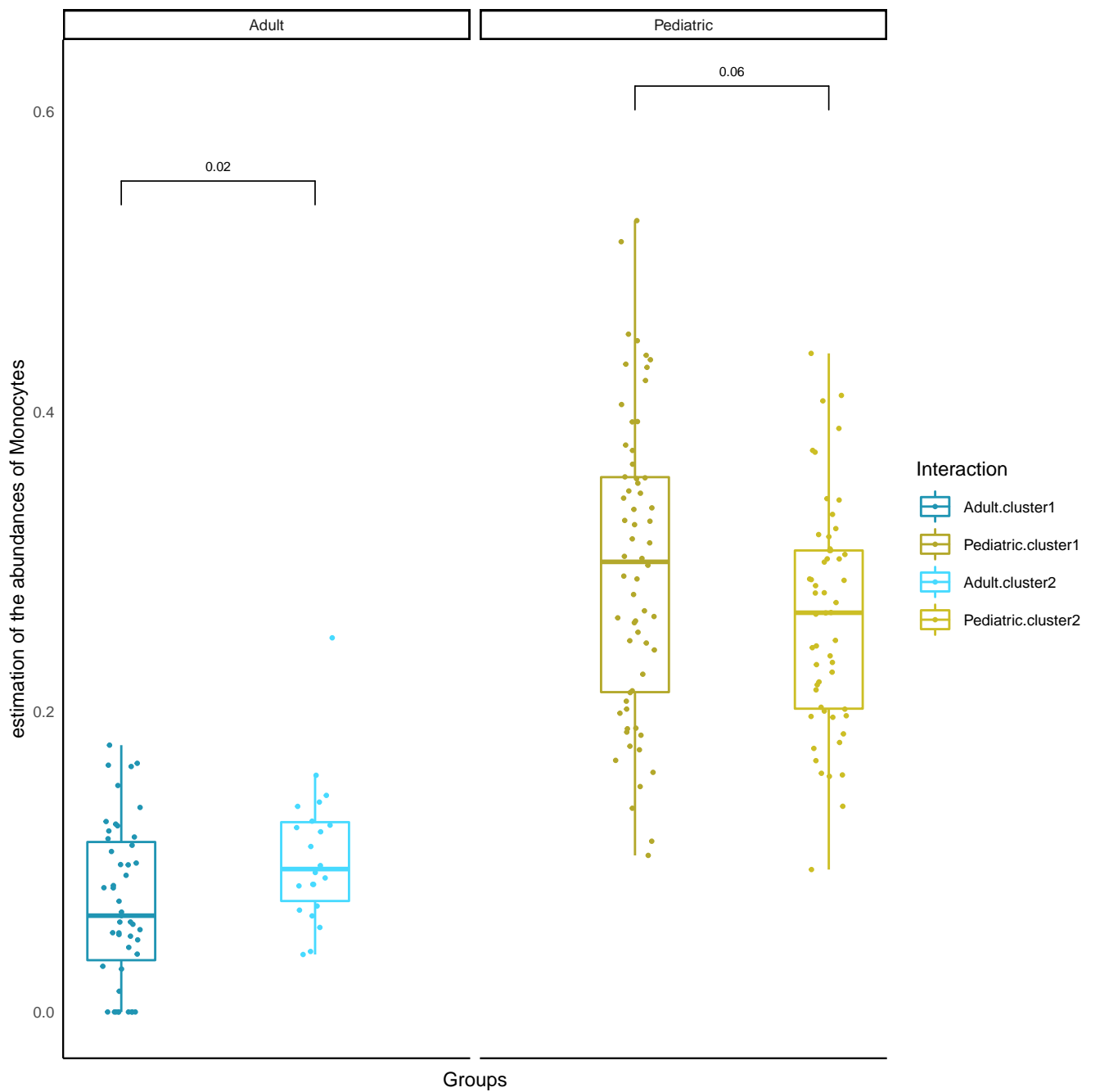

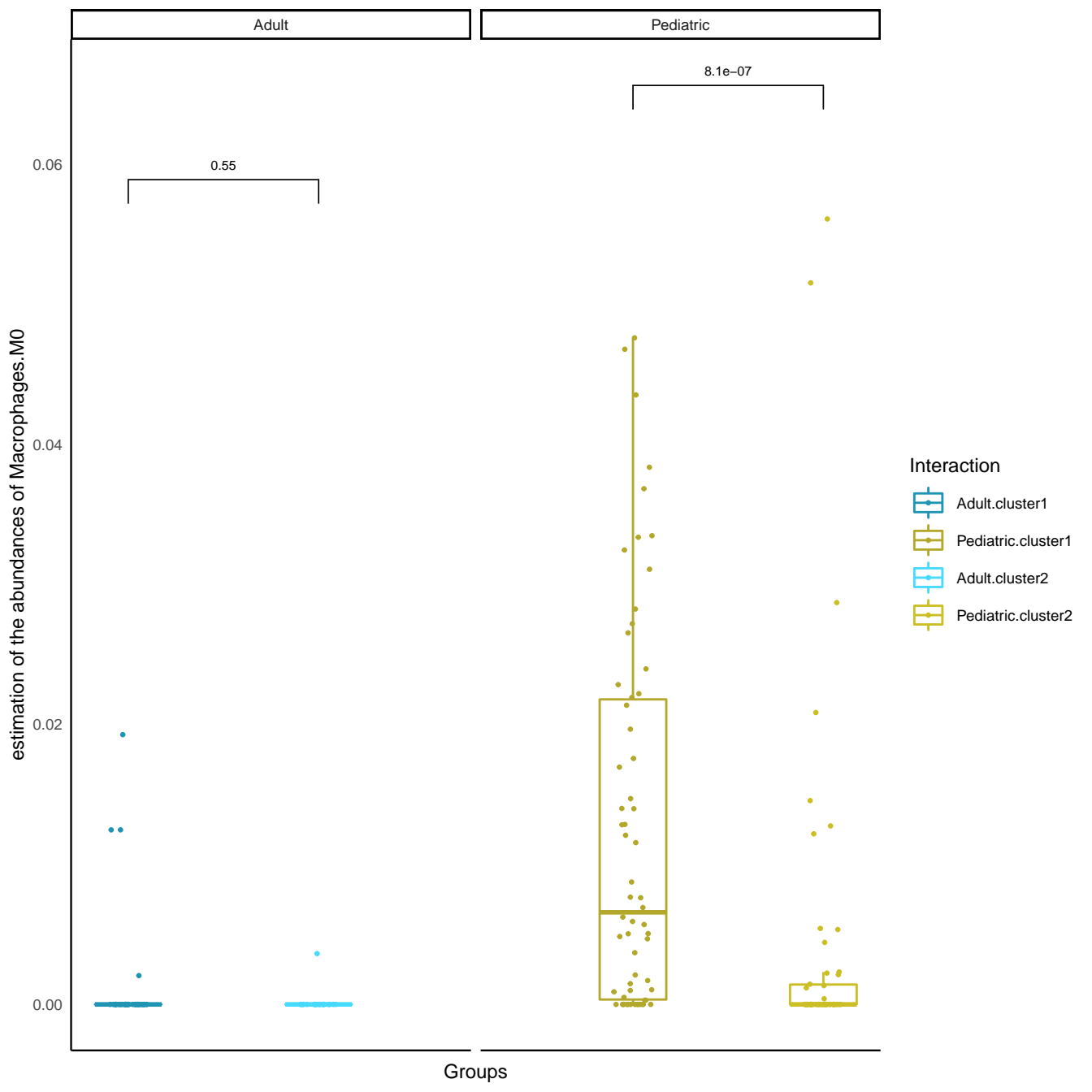

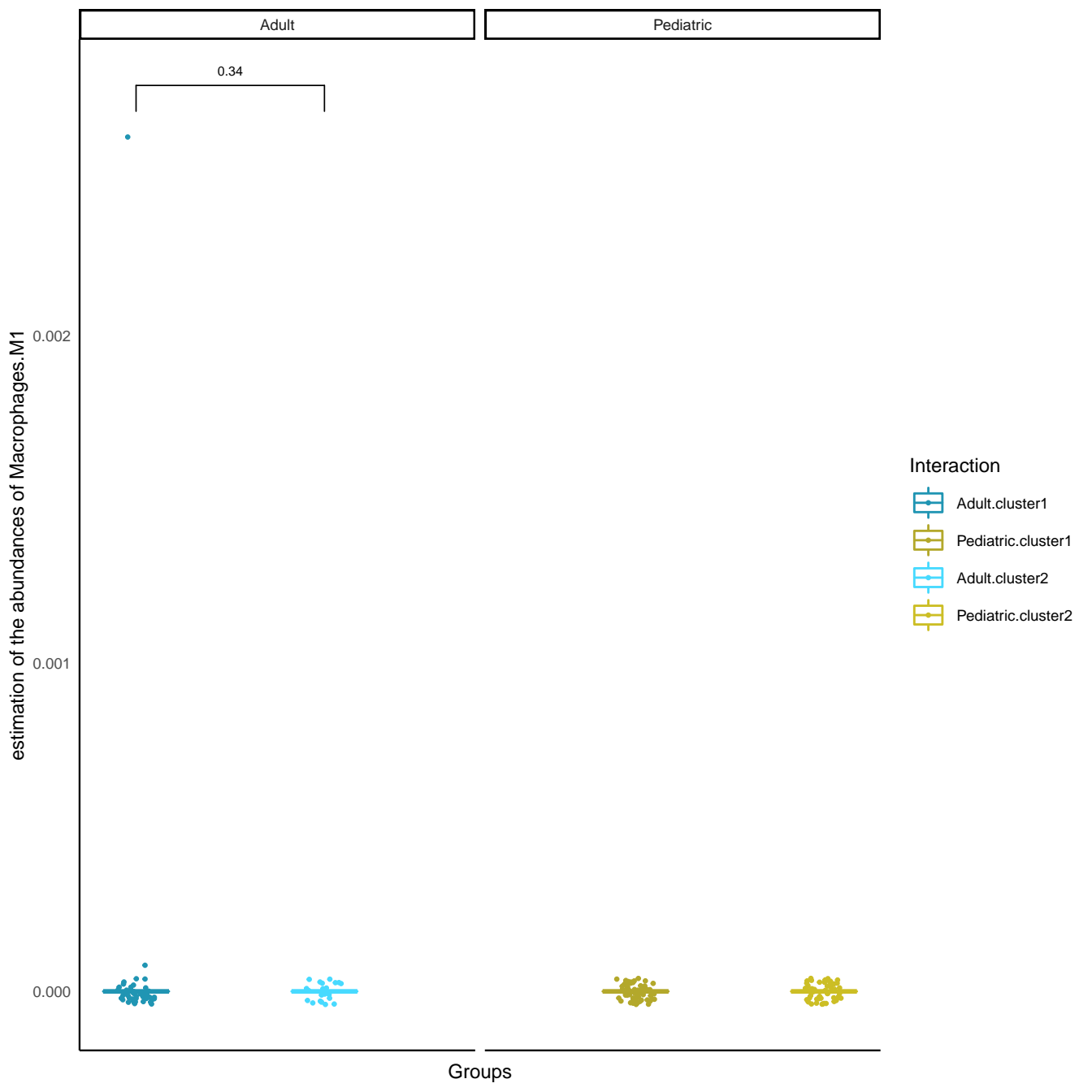

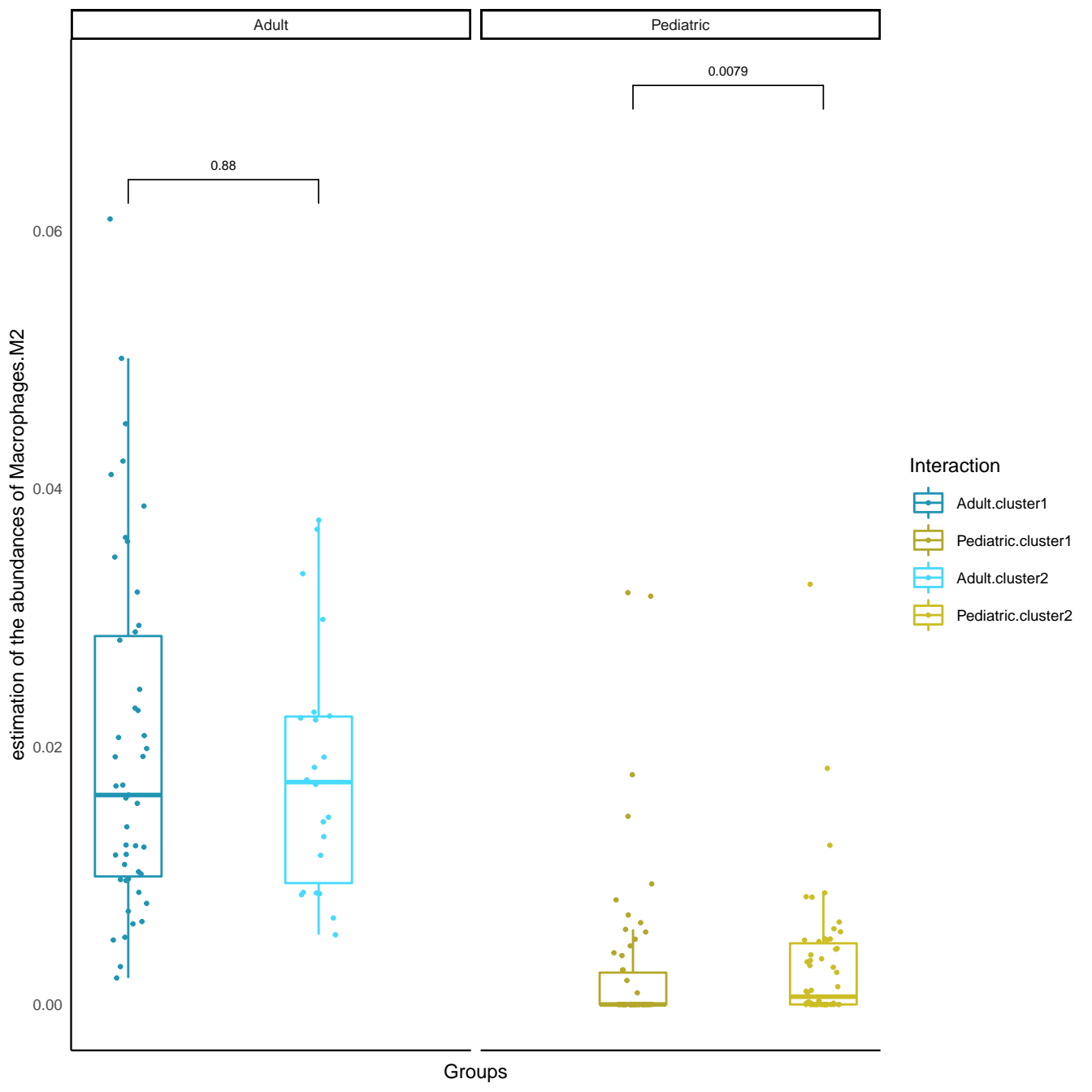

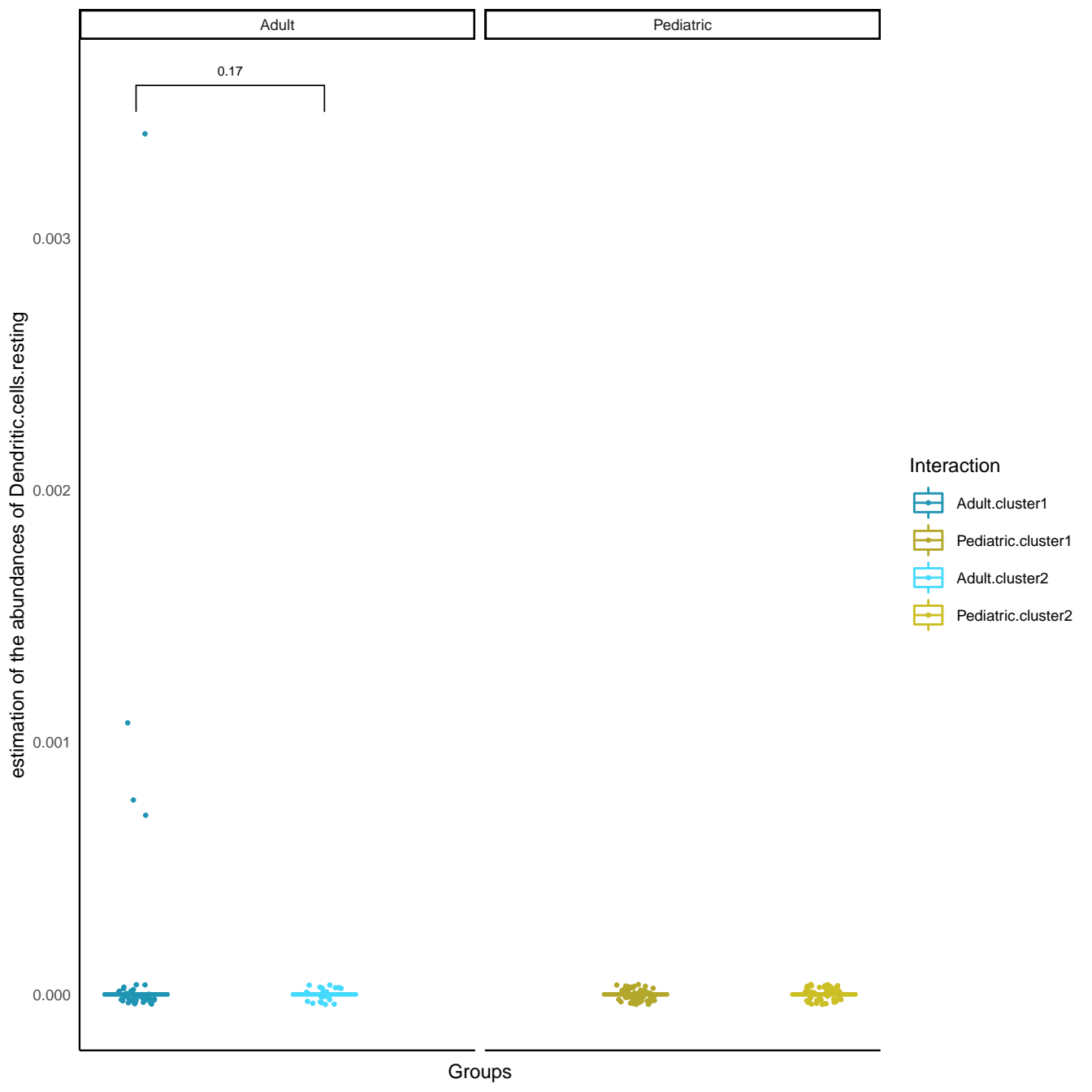

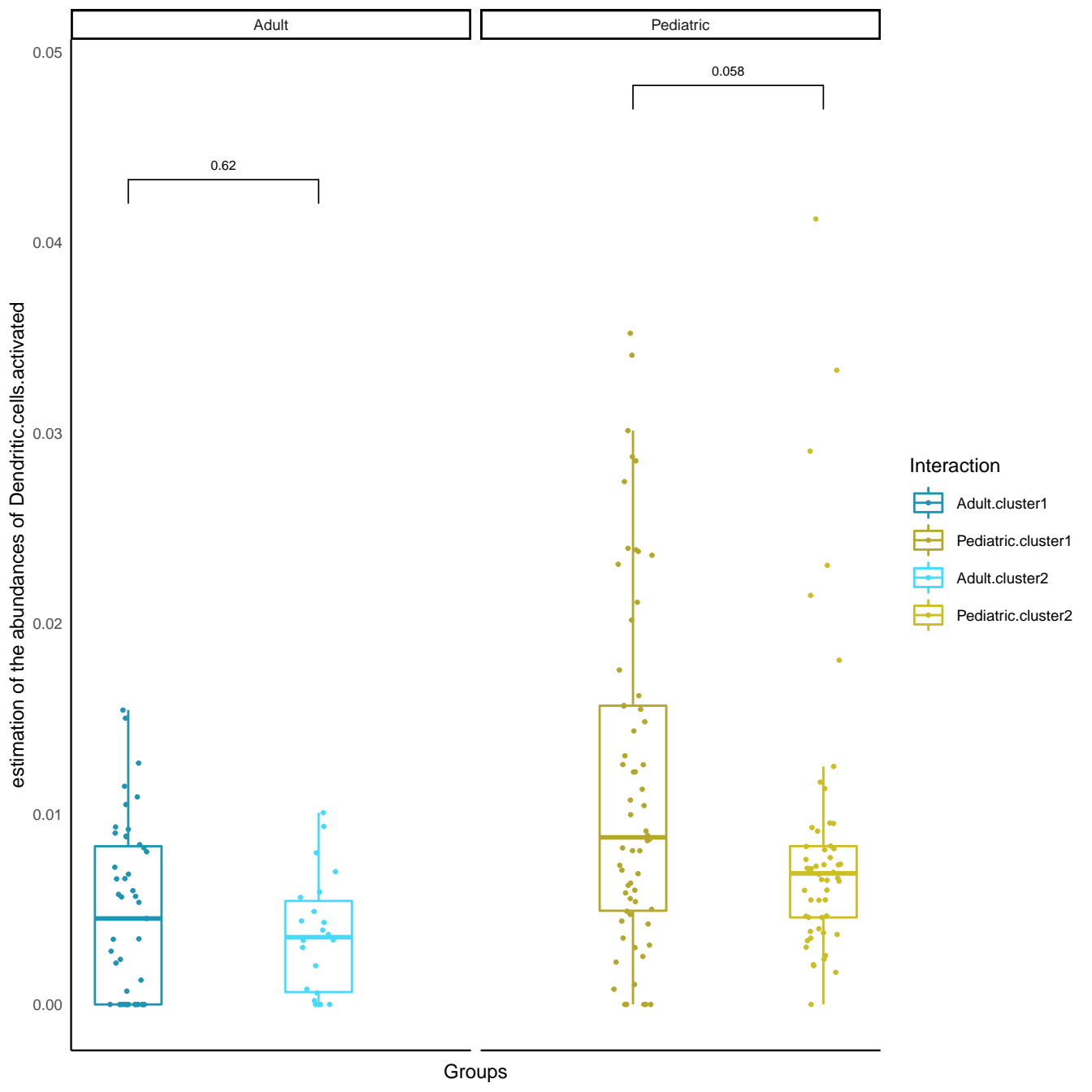

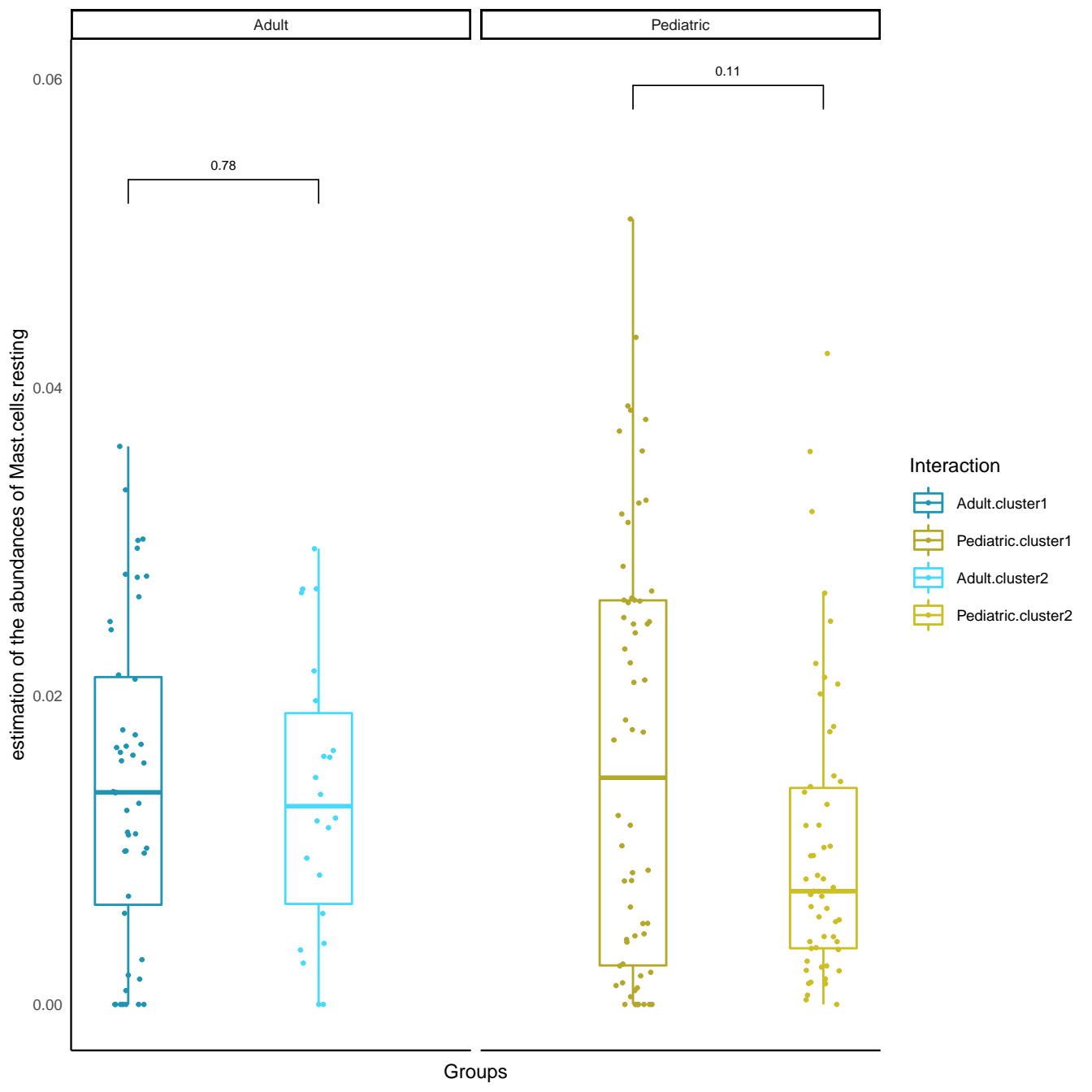

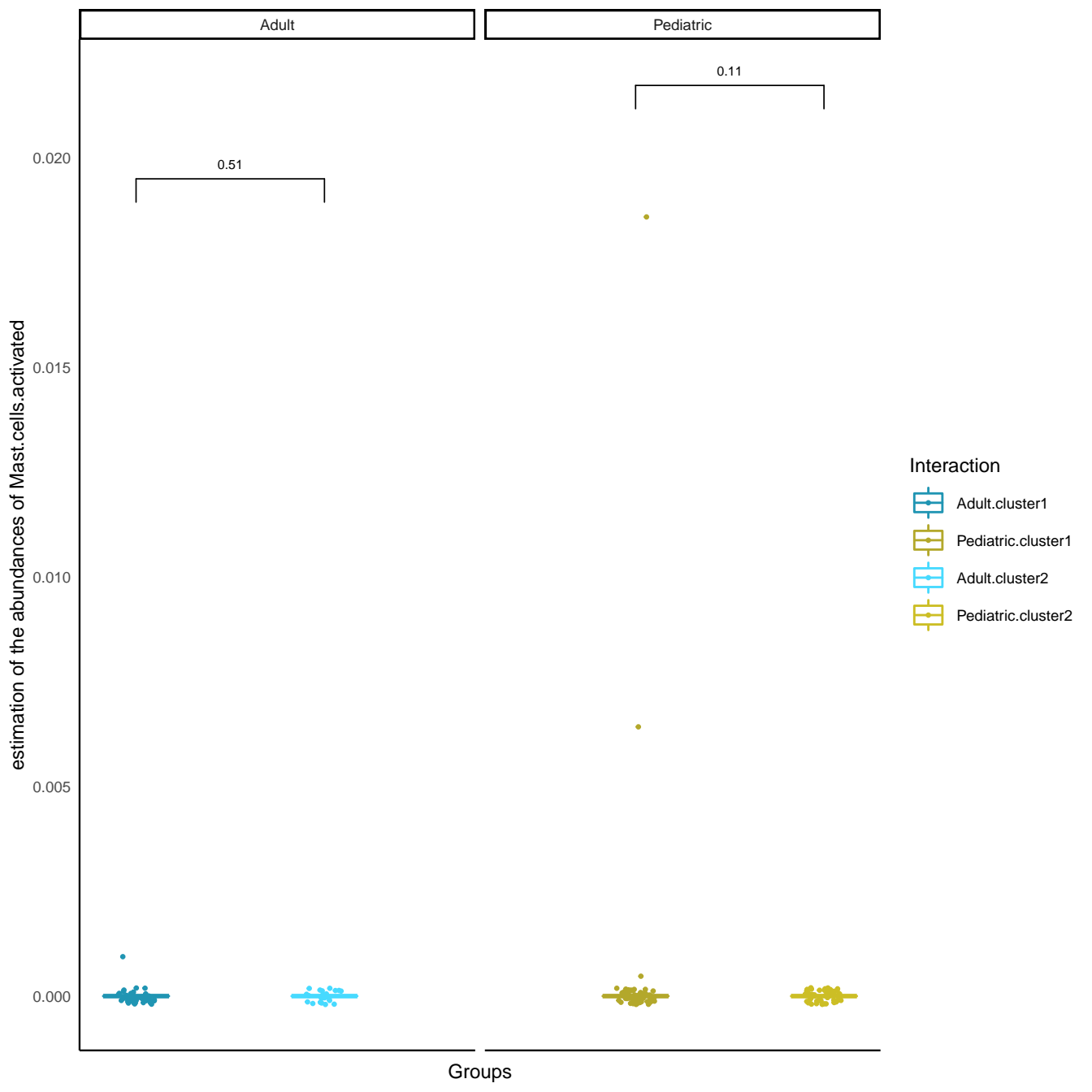

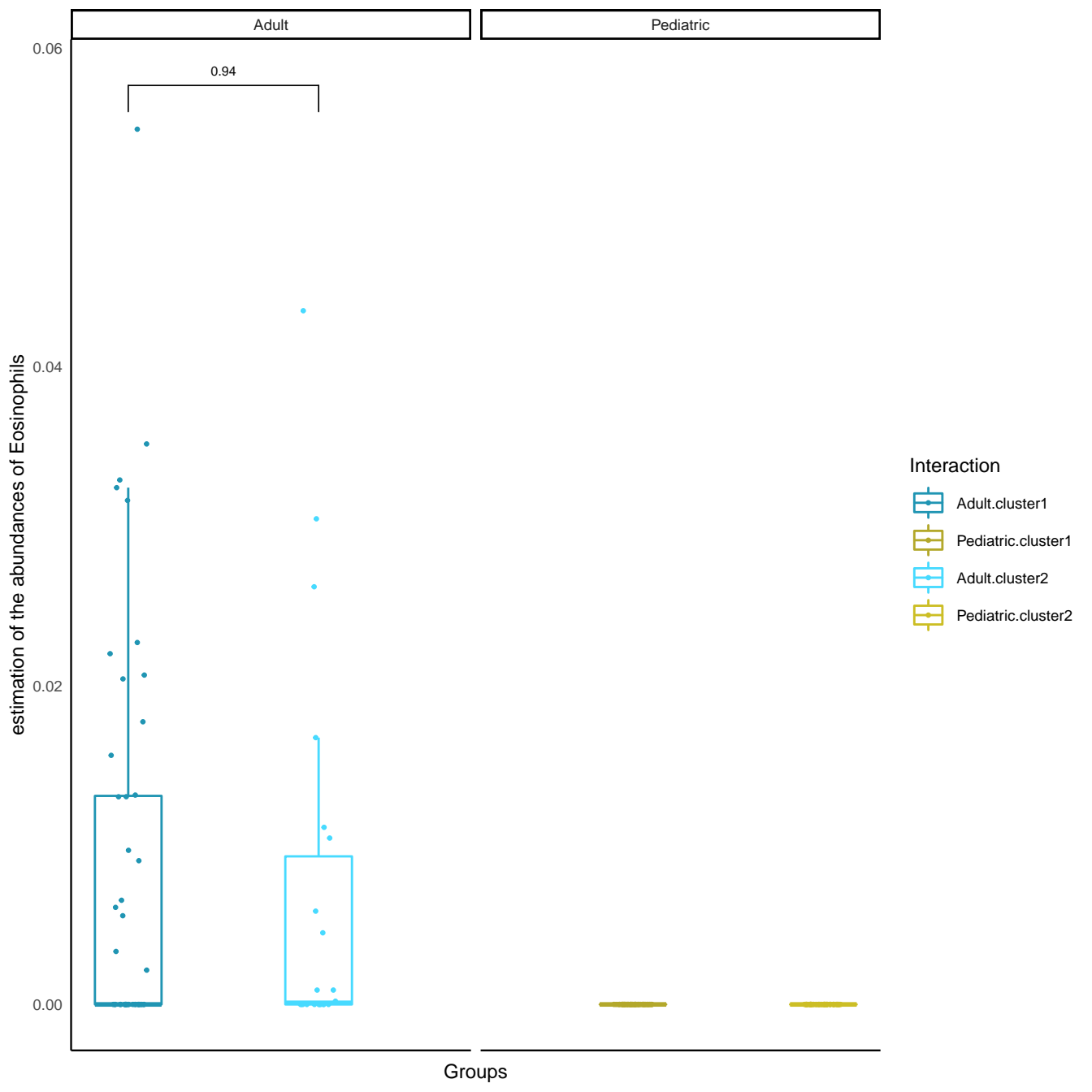

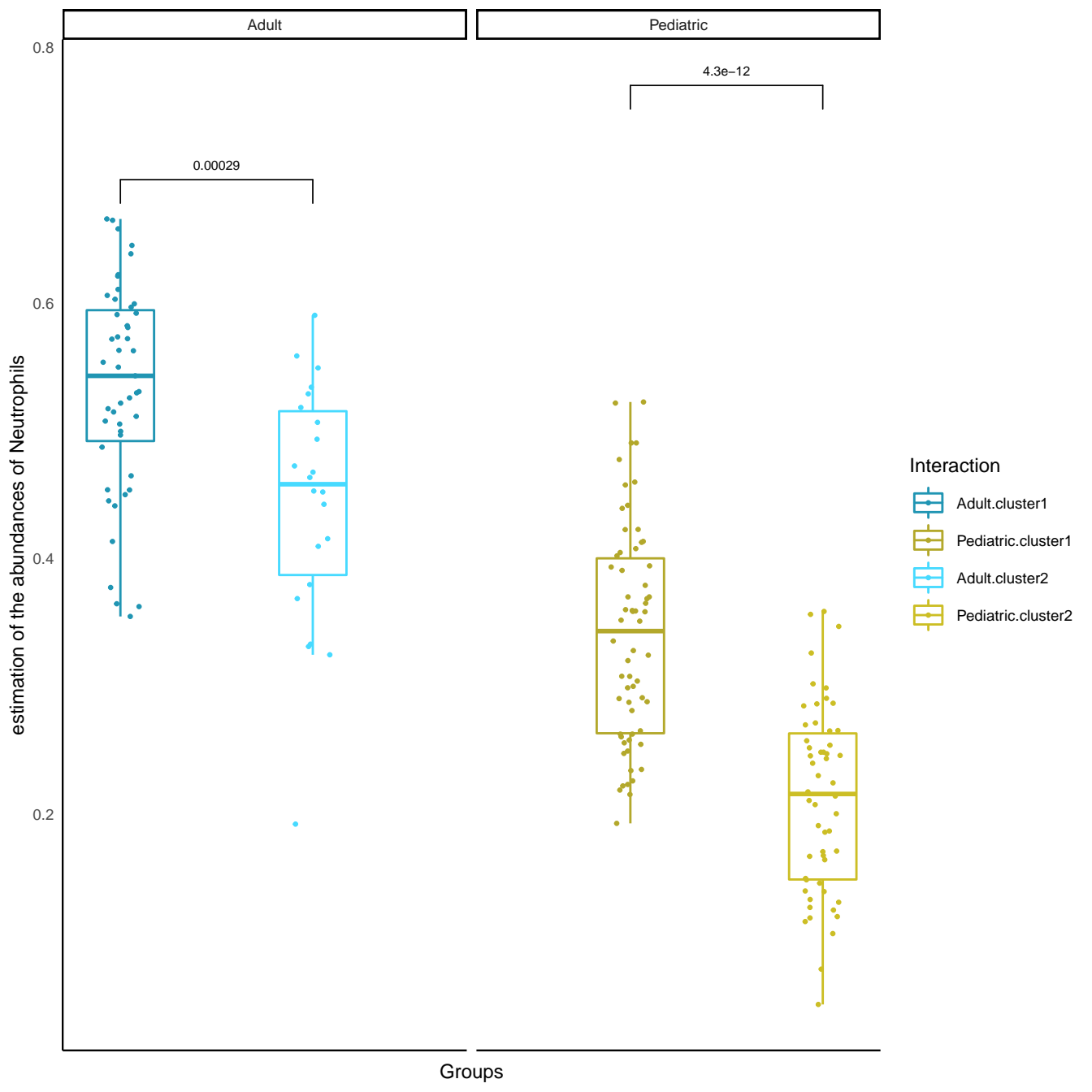

Supplement: Supplementary file 1 [file life-11-00299-s001.zip › Supplementary_Figure_1.pdf]

A

Adult Dataset

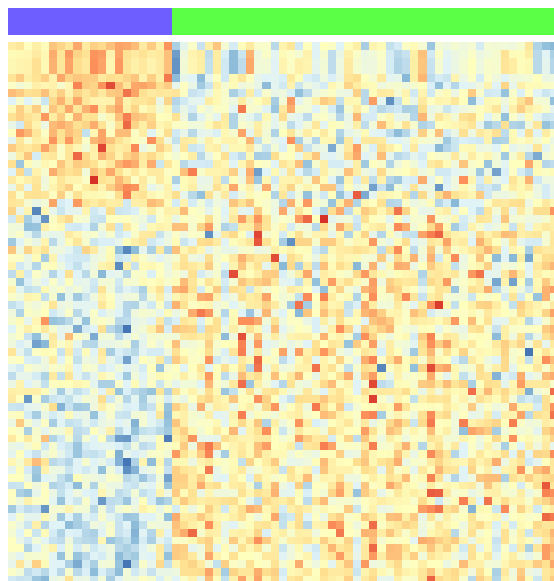

Pediatric Dataset

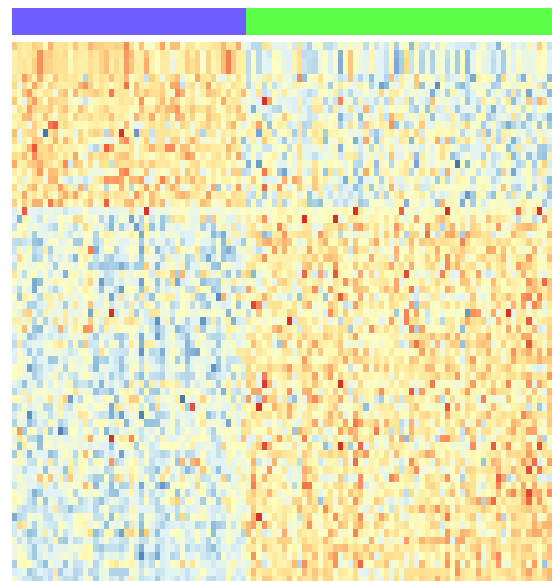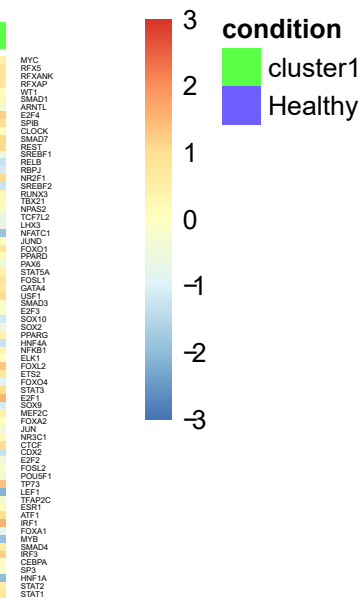

B

Adult Dataset

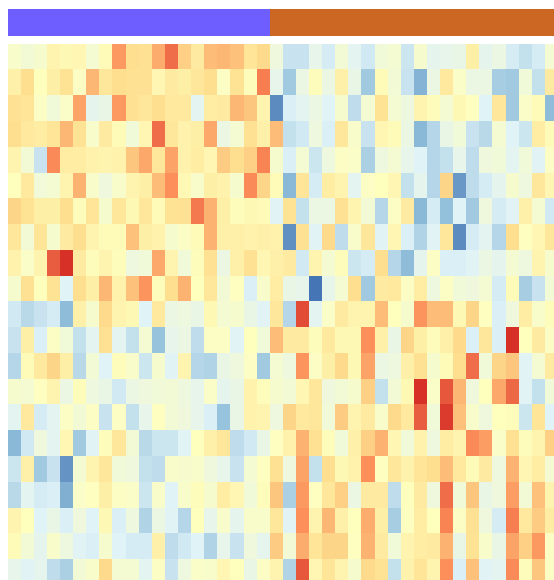

Pediatric Dataset

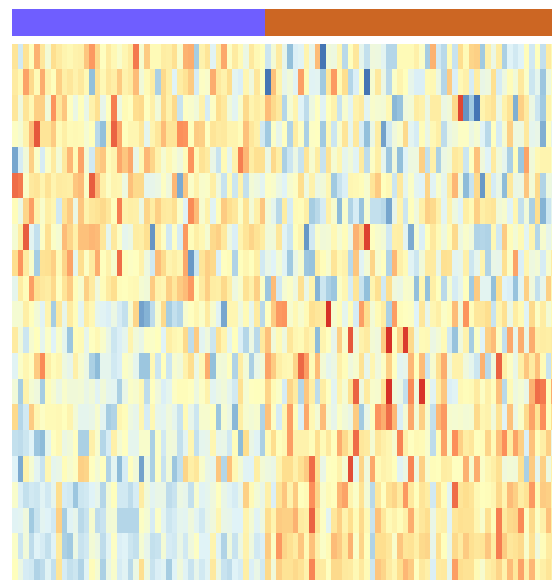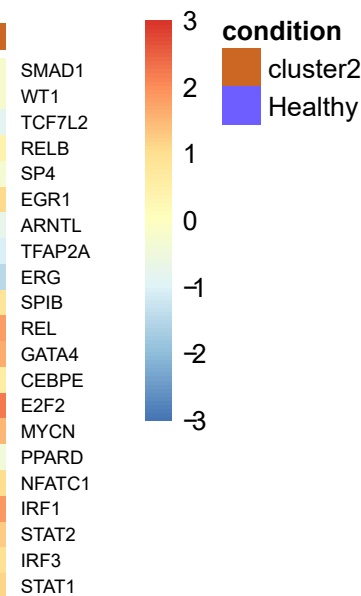

Supplement: Supplementary file 1 [file life-11-00299-s001.zip › Supplementary_Figure_3.pdf]
